# Heterogeneous transfusion risk linked to variant Creutzfeldt-Jakob disease in a non-human primate model

**DOI:** 10.1371/journal.ppat.1014769

**Published:** 2026-11-10

**Authors:** Jessica Rontard, Jacqueline Mikol, Florian Almela, Daisy Bougard, Christelle Jas-Duval, Olivier Andreoletti, Jérôme Delmotte, Mohammed-Amin Madoui, Jean-Philippe Deslys, Emmanuel E. Comoy

**Affiliations:** 1 Commissariat à l’Energie Atomique, DRF/Jacob/SEPIA, 92265 Fontenay-aux-Roses, France; 2 Etablissement Français du Sang, 34184 Montpellier, France; 3 UMR INRAe-ENVT 1225 IHAP, Ecole Nationale Vétérinaire de Toulouse, 31076 Toulouse, France; Colorado State University College of Veterinary Medicine and Biomedical Sciences, UNITED STATES OF AMERICA

## Abstract

Among the 178 cases of variant Creutzfeldt-Jakob disease (v-CJD) in the UK, three were reported between 2003 and 2007 to be linked to a probable transmission through blood transfusion. With no further primary or iatrogenic cases observed worldwide since 2016, several countries are lifting – or have already lifted - deferrals for blood donors who have lived in the UK or, more broadly, Europe, to reduce the threat of supply shortages. However, the high prevalence of silent infection according to abnormal PrP in lymphoid tissues (potentially 100 times higher than for clinical cases in the UK) remains a concern in assessing the potential iatrogenic risk associated with long-term silent carriers for a disease whose incubation periods can extend over decades. In the present study, after experimental intravenous contamination of cynomolgus macaques with v-CJD prions, a relevant model of the human disease, we observed a highly heterogeneous intra- and inter-individual distribution of apparent peripheral prion replication in lymphoid organs that appears to correspond largely to the prion load in blood, the highest levels of which are detectable by amplification-based detection assay. In addition, several primates have developed unexpected incomplete prion phenotypes that escape diagnosis by current conventional techniques and could be intravenously transmitted to mice in the absence of a functional immune system. The peculiar prion strains associated with these atypical phenotypes would involve peripheral mechanisms independent of the immune system, different from those of v-CJD. According to our observations, conventional lymphoid organ prevalence studies may underestimate the true extent of healthy v-CJD carriers, and blood transmission risk assessment should not be based on epidemiological data alone.

## Author summary

Variant Creutzfeldt-Jakob disease (v-CJD) is considered a transfusion-transmissible infectious agent. In the absence of cases since 2016, several countries are lifting geographical restrictions on blood donation to reduce supply shortages. However, appendix studies have revealed a high prevalence of silent infection that questions the iatrogenic risk associated with long-term carriers (incubations extend over decades). Here, intravenously-contaminated macaques exhibited a highly heterogeneous distribution of v-CJD prion replication in lymphoid organs corresponding largely to the prion load in blood, whose highest levels are detectable by amplification-based detection assay. In addition, several primates developed incomplete prion phenotypes that escape diagnosis by current conventional techniques and could be intravenously transmitted to immunodeficient mice: the corresponding cryptic prion strains would involve immune-independent mechanisms, unlike v-CJD. According to our observations, the heterogeneity of peripheral prion replication observed in experimentally-infected macaques questions the actual prevalence of asymptomatic v-CJD carriers and the assessment of blood transmission-related risks in the human population.

## Introduction

Prion diseases are incurable transmissible neurodegenerative disorders with long silent incubation periods extending up to half life expectancy (human cases arose almost 50 years post-exposure) [1,2], featuring accumulation of abnormal isoform of prion protein (PrP) in the Central Nervous System (CNS), tissue damage (spongiform change) and cellular lesions (astrogliosis and neuronal loss). Following peripheral administration, prions initially replicate silently in the follicles of lymphoid organs (lymph nodes (LN), spleen and gut-associated lymphoid tissue) before invading the CNS [3–5].

Over the past decades, European consumers were massively exposed to Bovine Spongiform Encephalopathy (BSE) agent through contaminated food [6]. Fortunately, the resulting number of v-CJD cases [7] is so far limited to 230 primary cases worldwide, suggesting a high cattle-to-human species barrier [8]. However, a second risk for public health rapidly emerged: three v-CJD cases were linked to contamination by Red Blood Cells (RBCs) transfusion and a fourth individual devoid of v-CJD symptoms was post-mortem identified [9]. A fifth asymptomatic patient was also described after a possible contamination through plasma-derived products [10]. Precautionary measures, including donor deferral [11] and systematic leukoreduction [12] were implemented to limit this mode of contamination. Over the last decade, no further transfusion-related cases have been reported: subsequently, several countries lifted this deferral due to its economic cost and the shortage of blood donations, and others are following suit [13–15].

Conversely, the safety of blood towards v-CJD transmission remains debatable. Abnormal PrP deposition was identified in 16 out of 32,441 appendectomy specimens sampled between 2000 and 2012 [16]: the resulting prevalence of clinically-silent individuals infected with v-CJD is estimated to be 1/2000, approximately 200 times higher than detected clinical patients, among Britons exposed to the BSE epizootic. Nevertheless, similar prevalence was estimated among people born shortly after BSE ban (1996–2000), and also in 10–38 years-old people that were sampled before the report of first BSE cases (1977–1979), whereas no positivity was observed in other cohorts (sampled before 1976 or born after 2001): BSE exposure would have been more widespread than previously thought, i.e., both before and after the recognized period, from 1986 to 1996. Birth cohort which has been considered as an exclusive reliable inclusion criterion for blood donation, may have missed a fraction of infected donors [17]. These healthy carriers might be seen as potential blood or tissue donors [18], and their progression to infection and ability to transmit the disease through blood donations remain elusive, even 30 years after the peak of human exposure [1,2]. Various promising ante-mortem tests were subsequently reported as an additional measure to improve transfusion safety towards prions [19–22]: most of them can detect abnormal PrP before the onset of clinical signs, but none is to date validated for routine screening of blood donors.

Experimental infection of cynomolgus macaques with BSE or v-CJD prions is considered a relevant model of human situation towards primary (cattle-to-human) and secondary (inter-human) risk of transmission respectively [23–27], and their blood is proposed as a collection to validate those diagnostic test candidates [28]. Notably, we and others have shown that transfusion of infected blood products transmitted v-CJD in macaques [28–30] with volumes proportional to those used in human transfusion. In parallel, we described in several transfused macaques a different pathological entity based on spinal cord and brainstem involvement, which proved to be a distinct phenotypic expression of prion infection [30,31]: these animals exhibited an exclusive expression of cleaved C1 fragment of physiological PrP in their spinal cord, concomitantly to the absence of abnormal protease-resistant PrP (PrP^res^) in their CNS samples [32]. We previously described such an absence of PrP^res^ in the frame of incomplete prion phenotypes occurring in C57Bl/6 mice primarily exposed to BSE [33], and in Swiss mice transfused with v-CJD-derived blood [30]. In this latter model, transfused Swiss mice exhibited fatal neurological signs associated with histological lesions (spongiform changes and neuronal lesions, but without PrP deposition) restricted either to their brain, brainstem or spinal cord, and therefore we named these incomplete prion phenotypes as cerebral, bulbar or spinal phenotypes respectively. According to our observations, this myelopathic syndrome in macaques and incomplete phenotypes in conventional (non-transgenic) mice would occur under non-optimal conditions of contamination which would rather lead to subclinical infections in the sheep model of transfusion [34]. These different issues of infection in these three experimental animal models question the amplitude of transfusional risk in the human population.

The factors determining blood infectivity remain elusive: notably, the window of the asymptomatic period with a real transfusion risk is unknown to date. In our first attempt to determine the conditions for such efficient transmission, we analyzed the presence of abnormal PrP in the peripheral lymphoid organs (lymph nodes and spleen) and blood of several macaques intravenously exposed to BSE/v-CJD infection in the frame of previously published experiments [24,29,30], and their ability to transmit disease by blood.

## Results

### Intra- and inter-individual heterogeneity of abnormal PrP distribution in lymphoid organs

We included here 27 macaques intravenously exposed to cerebral and blood products derived from BSE or v-CJD-exposed donors, issued from six independent studies whose details are provided in Table 1 (BSE or v-CJD origin of infection is detailed in this table, but the generic term of BSE/v-CJD will be kept throughout the text for easy reading). Among them, 17 animals developed clinical BSE/v-CJD 2–5 years post-exposure with specific clinical and lesional features [23,35]. The 10 other animals developed the myelopathic syndrome devoid of detectable abnormal PrP as previously extensively described [30], with demyelination and pseudo necrotic lesions focused on the spinal cord, brainstem and optic nerves. For each lymph node sampled from these 27 animals, the numbers of positive and total follicles were recorded (S1 Table) and compared to the presence of PrP^res^ in their spleen.

**Table 1:** List of primates included in the present study (most of them are detailed in [30]).

Primate	Inoculum	Dose	Incubation (months)	Clinical period (months)	Diagnosis	Identity in Ref. 30
Study #1: BSE-infected macaque brain IV inoculation (crude homogenate)						
#01	Brain	0.4 mg	30	3	BSE	D11
#02	Brain	0.04 mg	47	4	BSE	D12
Study #2: BSE-infected macaque brain IV inoculation (clarified homogenate)						
#03	Clarified brain	10 mg	42.5	9	BSE	D19
#04	Clarified brain	10 mg	45.5	12	BSE	D17
#05	Clarified brain	10 mg	34.5	3	BSE	D18
#06	Clarified brain	100 mg	33	2.5	BSE	D16
#07	Clarified brain	100 mg	35	9	BSE	D15
Study #3: v-CJD-infected macaque brain IV inoculation (clarified homogenate)						
#08	Clarified brain	10 mg	42	3	Myelopathy	D7
#09	Clarified brain	10 mg	47	3.5	v-CJD	D6
#10	Clarified brain	100 mg	33	7	v-CJD	D5
#11	Clarified brain	100 mg	33	5	v-CJD	D4
#12	Clarified brain	100 mg	32	6	v-CJD	D3
#13	Clarified brain	100 mg	29	8	v-CJD	D2
Study #4: BSE-infected macaques blood transfusion (pool derived from #3, #4, #5, #6, #7)						
#14	Whole blood	40 ml	50	2	BSE	R15
#15	Leukodepleted RBCC	27 ml	30	2.5	Myelopathy	R16
#16	Leukodepleted RBCC	27 ml	30	2	Myelopathy	R17
Study #5: v-CJD-infected macaques blood transfusion (pool derived from #8, #9, #10, #11, #12, #13)						
#17	Whole blood	40 ml	62	7	v-CJD	R2
#18	Leukodepleted RBCC	24 ml	60	1	v-CJD	R4
#19	RBCC	24 ml	43	5	v-CJD	R3
#20	RBCC	24 ml	40	3.5	Myelopathy	R5
Study #6: individual blood transfusions						
#21	Whole blood BSE-infected macaque	20 ml	32	4.5	Myelopathy	R18
#22	Whole blood BSE-infected macaque	40 ml	141	5	Myelopathy	R8
#23	Leukodepleted blood BSE-infected macaque	40 ml	139	30	Myelopathy	R10
#24	Whole blood v-CJD-infected macaque	40 ml	62	4.5	v-CJD	R1
#25	Plasma + buffy coat v-CJD-infected patient	40 ml	65.5	6	Myelopathy	R6
#26	Whole blood v-CJD-infected macaque	40 ml	29	8	Myelopathy	
#27	Plasma v-CJD-infected macaque	23 ml	23	0.5	Myelopathy	

The distribution of abnormal PrP in the peripheral tissues of the 17 clinically-affected BSE/v-CJD animals was widely heterogeneous (Fig 1 and Fig 2a), ranking from an absence of detection (primate #5, Fig 1a) or few (primate #24, Fig 1b) apparent foci of accumulation in spleen and LN, to the presence of abnormal PrP in all the tested lymphoid organs (#10 and #19, Fig 1c). Noteworthy, four animals (#10, #11, #12 and #13) identically exposed and displaying similar incubation periods (29–33 months) exhibited a marked inter-individual variability.

**Fig 1:**
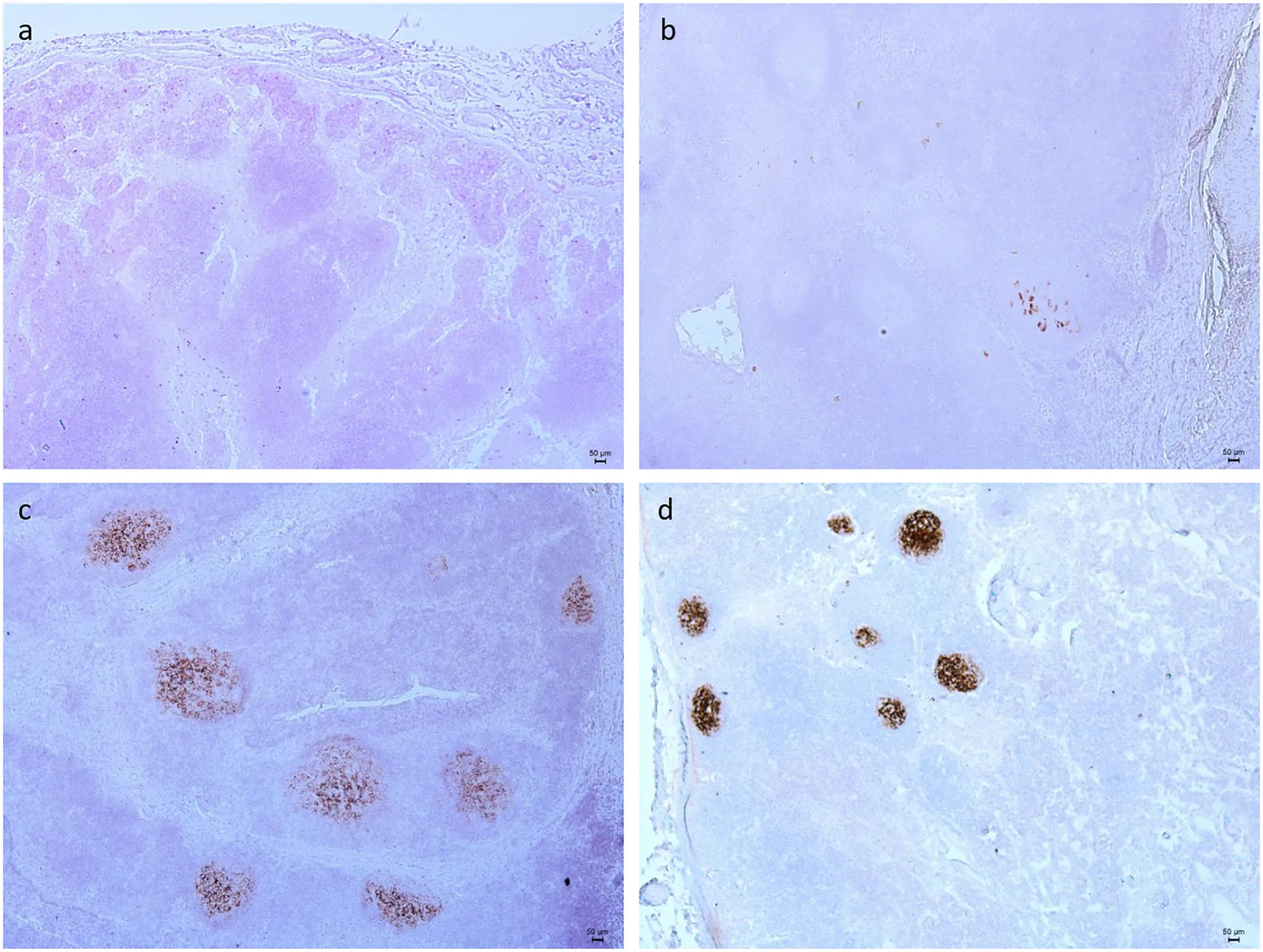
Immunodetection of the abnormal prion protein, using 3F4 anti-PrP antibody, in peripheral LN of macaques exposed to BSE/v-CJD infected inocula. In v-CJD macaques, the ratios of positive follicles in LN ranged from (a) no positive follicle (mesenteric lymph node, macaque #5) or (b) few positive follicles (submaxillary lymph node, macaque #24) to (c) all the follicles to be positive (inguinal lymph node, macaque #19). (d) In myelopathic macaque #20, a strong PrP immunoreactivity was observed (axillary lymph node).

**Fig 2:**
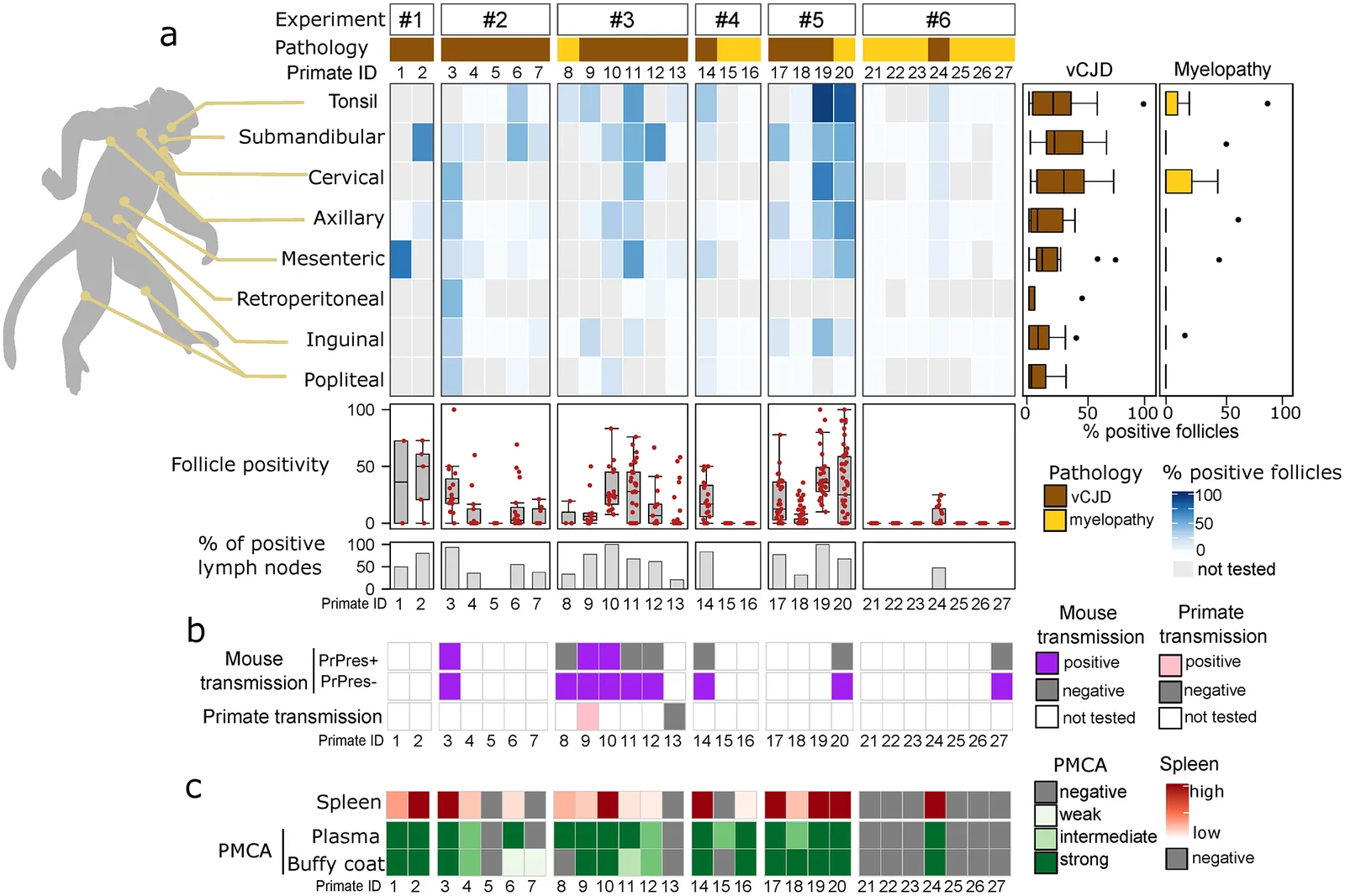
Apparent peripheral distribution of prions in primates experimentally infected through the intravenous route within the frame of 6 independent studies (numbered #1 to #6 in Table 1). For the 27 primates (17 BSE/v-CJD and 10 myelopathic primates, pathologies respectively identified in brown and yellow; details of contamination are provided in Table 1), **a)** the percentage of positive follicles was scored for each lymph node (tonsils, submandibular, cervical, axillary, mesenteric, retroperitoneal, inguinal and popliteal) sampled (red points). Individual and merged results are presented according to anatomical distribution, animal and pathology. For each animal, the percentage of positive lymph nodes (harboring at least one positive follicle) is also mentioned. **b)** Transmissions in Swiss mice (PrP^res+^: v-CJD transmission; PrP^res-^: occurrence of alternative phenotypes of prion diseases devoid of apparent accumulation of PrP^res^). and one-to-one transfusion in primate. Positive transmissions are mentioned in purple or pink respectively, whereas negative transmission is mentioned in gray (white: not performed). **c)** An estimation of the amount of PrP^res^ detected in spleen by ELISA was provided as a color gradient – gray = negative (OD < limit of detection). The results of PMCA analysis of plasma and buffy coat (BC) sampled at the terminal stage are provided as a color code on the basis of duplicates: strong = 2/2 positive results after 2 (plasma) or 3 (BC) rounds of PMCA; intermediate = 2/2 positive results after 3 (plasma) or 4 (BC) rounds of PMCA; weak = 2/2 positive or 1/2 low positive results after 4 (plasma) or 5 (BC) rounds of PMCA; negative = 2/2 negative results after 4 (plasma) or 5 (BC) rounds of PMCA. This scoring is summarized in **S1 Fig**, which also exhibits representative results of PMCA amplification.

This inter-individual variability in follicular PrP positivity was supported by an intraclass correlation coefficient (ICC) of 0.313 and by the strong improvement of model fit when primate identity was included as a random effect (χ^2^ = 1067.91, df = 1, p = 3.12 × 10 ^−234^). The influence of lymph-node anatomy, as illustrated in S2 Fig, was evaluated after merging left and right homologous regions within each primate. A beta-binomial mixed model with region as a fixed effect and primate identity as a random effect showed a significant regional effect (χ^2^ = 28.9, df = 7, p = 1.5 × 10 ^−4^). PrP positivity was highest in tonsils (32.4%), followed by submandibular (24.0%), cervical (20.1%), and mesenteric nodes (15.2%). Tukey-adjusted comparisons (p < 0.05) placed tonsils in the highest group, axillary/inguinal/popliteal/retroperitoneal nodes in the lowest, and submandibular, cervical, and mesenteric nodes as intermediate. Taking all these BSE/v-CJD animals together, abnormal PrP deposition was detected in 17% of the lymphoid follicles observed (1,740/10,054), themselves distributed in 56% (201/355) of the lymphoid organs.

Within these positive LN, the percentage of positive follicles was very heterogeneous (Table 2), with only 16% (33/201) exhibiting PrP accumulation in more than half of the observed follicles.

**Table 2:** Irregular distribution of positive follicles into peripheral lymphoid organs. For primates, the percentage of positive follicles in each lymph node analyzed was determined and grouped in three categories (limited: < 25% positive follicles; average 25-60%; widespread > 61%) similar to those described in the appendix study survey in humans [16].

Distribution	% of positive follicles	Experimentally-exposed v-CJD primates		Human appendices	
		N lymph nodes	%	N appendices	%
Limited	2% - 24%	101	50%	5	31%
Average	25% - 60%	83	41%	7	44%
Wide	61% - 100%	17	9%	4	25%

Among the 10 myelopathic macaques, 7 had no detectable accumulation of abnormal PrP, while two exhibited weak positivity in only one of their lymphoid organs (tonsil - 7 positive among 36 follicles - of macaque #8 and spleen of macaque #16). The tenth macaque (#20, Fig 1d) exhibited high levels of peripheral replication within its spleen and two-thirds (33/49) of its LN, which is ultimately a higher level than in most of the BSE/v-CJD macaques. It is to note that this animal was exposed to the same inoculum (RBC concentrates) as the v-CJD macaque with the highest levels of peripheral replication (#19).

### Blood infectivity in BSE/v-CJD -exposed primates

The infectivity of blood products derived from six BSE/v-CJD and three myelopathic macaques sampled at their terminal stage was assessed through IV (plasma) or IC (buffy coat) inoculation in Swiss mice (Fig 2b, detailed transmission in S2 Table). Limited transmission of a v-CJD profile (noted as PrP^res+^) was observed in recipient mice from only three BSE/v-CJD macaques (#3, #9 and #10), which were among those with the highest levels of abnormal PrP peripheral accumulation (spleen and level of PMCA amplification in blood samples). Conversely, the plasma samples from all nine donors induced incomplete prion disease phenotypes as previously described [30] and compiled here as PrP^res-^ prion phenotypes in around 10% (4–15% for each individual macaque) of recipient mice (24/239) independently of the level of peripheral accumulation in donors (details provided in S2 Table). Incomplete prion phenotypes were also observed in mice exposed to buffy coat samples, but at a much lower rate.

In the frame of one-to-one transfusion experiments between macaques (Fig 2b), plasma (equivalent to 30 ml of whole blood) derived from a “high-replication” (PrP^res^ in spleen and in more than half of analysed lymph nodes) macaque #9 at the terminal stage transmitted v-CJD 68 months post-inoculation, whereas two macaques exposed to similar amounts of plasma derived from the “low-replication” macaque #13 (spleen negative and only 68/999 positive follicles corresponding to 9/43 positive LN) are still asymptomatic close to 15 years months post-inoculation.

With two pools of blood samples including the presence of at least one “high-replication” donor (macaques #3 and #10 respectively), the presence of peripheral abnormal PrP (i.e., protease-resistant PrP in spleen detected by ELISA, PrP accumulation visualized in follicles and/or abnormal seeding activity in spleen or blood samples) confirmed that the infection was transmitted to all the 7 recipient animals after transfusion of the different blood-derived products (S3 Fig); however, four of these recipient animals developed a clinical v-CJD phenotype whereas the three other ones exhibited a myelopathic syndrome without any obvious correlation with peripheral expression of infection. It is noteworthy that myelopathic primates #8 and #20 exhibited PrP seeding activity in their spleen and spinal cord but not in their cerebral samples.

### Detection of abnormal PrP seeding activity in blood samples with PMCA techniques

We sampled the blood from these macaques throughout the course of their diseases, and corresponding plasma and buffy coat fractions were assessed using two distinct, dedicated optimized PMCA techniques (detailed results and protocols are provided in S3 Table and S4 Table respectively). In parallel, control samples derived from 12 healthy macaques were all tested negative. We first focused on the samples collected during the clinical phase for a potential correlation between the presence of seeding activity in blood fractions and the observed heterogeneity of abnormal PrP distribution in the lymphoid organs (Fig 2c), using two methods with similar limits of detection [36].

In BSE/v-CJD macaques, positive seeding activity was detected in 82% (14/17 primates) and 88% (15/17 primates) of plasma and buffy coat samples respectively. In the three macaques displaying either negative (#5 and #13) or inconsistent positive (#7, weak positive in buffy coat but negative in plasma) results in PMCA reactions seeded with blood fractions, no abnormal PrP accumulation was measured in their spleen, and they were either negative or showed low levels of accumulation in a few specific lymph nodes by IHC. Intermediate amounts of abnormal PrP were observed in blood samples derived from five BSE/v-CJD macaques (#4, #6, #11, #12 and #18) which all exhibited limited amounts of splenic PrP^res^^,^ whereas both plasma and buffy coat were detected as highly positive for the nine other animals. This included three blood samples derived from primates #3, #9 and #10 that also transmitted v-CJD by intravenous or intracerebral routes in the mouse bioassay. These results suggest, as expected, a correlation between the global amounts of abnormal PrP in lymphoid organs and the seeding potential in blood: Spearman correlation analysis identified a strong positive association between spleen PrP accumulation, PMCA positivity, and the percentage of PrP-positive follicles (ρ > 0.7, *P* < 0.01), indicating that these markers reflect a common axis of peripheral prion replication (Fig 3a).

**Fig 3:**
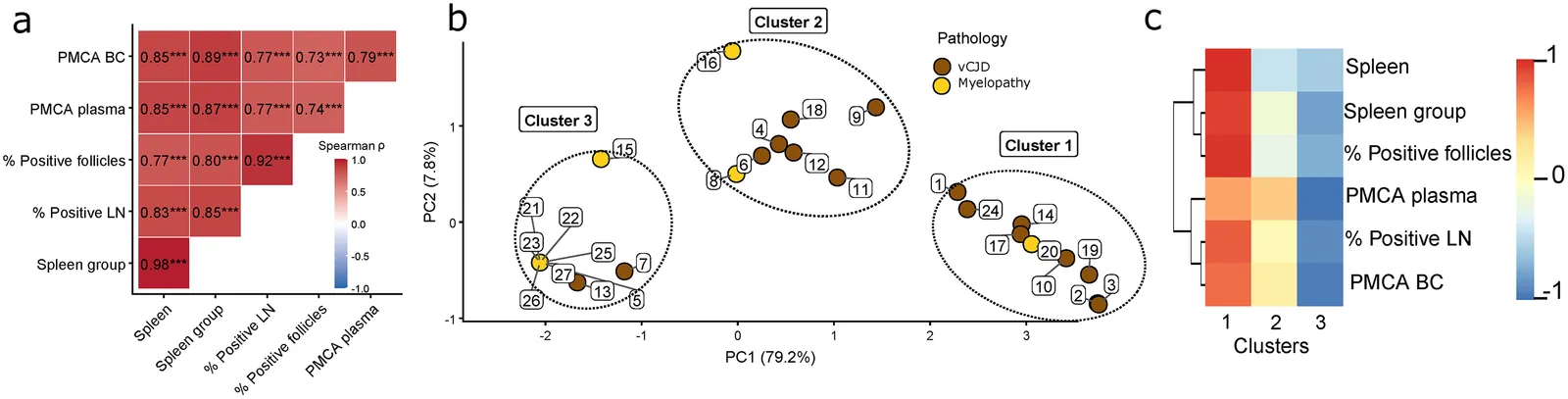
a) Upper triangular heatmap of pairwise Spearman correlation coefficients (ρ) between peripheral prion replication variables. Triple asterisks (***) indicate significant correlations, *P* < 0.001. b) Principal component analysis (PCA) of macaques based on peripheral prion replication variables. Individuals are coloured according to pathology, and dashed lines delineate the three clusters identified by k-means clustering. c) Heatmap showing the z-score values of each variable across the three clusters.

Among myelopathic primates, blood fraction samples from four macaques (40%) were detected as positive by PMCA, including the three primates (#8, #16, and #20) who exhibited peripheral accumulation in lymphoid organs, and for which at least plasma is highly positive. The fourth macaque (#15), devoid of any apparent peripheral accumulation, exhibited positivity only in plasma: this macaque was exposed to the same inoculum as primate #16 and develop myelopathy after similar incubation periods.

The samples collected along the silent incubation periods of 10 of these macaques were also tested by PMCA (Fig 4). When high levels of seeding activity were detected at the terminal stage of the disease (#10 #14, #17, and #19), all the samples collected during the second phase of the incubation period were also highly positive, i.e., close to two years before the onset of the clinical signs for some animals. When low levels of seeding activity were detected during the clinical phase (#4 and #12), samples collected during incubation periods were less reactive, and no seeding activity was observed in the primate #5 devoid of any detectable peripheral replication. Taken altogether, the seeding activity in blood products increases during the incubation period but can be inconstant when peripheral replication is limited. It is worth highlighting that the two PMCA techniques used here on BC or plasma samples were able to detect seeding activity during the first quartile of the incubation periods onwards, as soon as 6–9 months post-infection.

**Fig 4:**
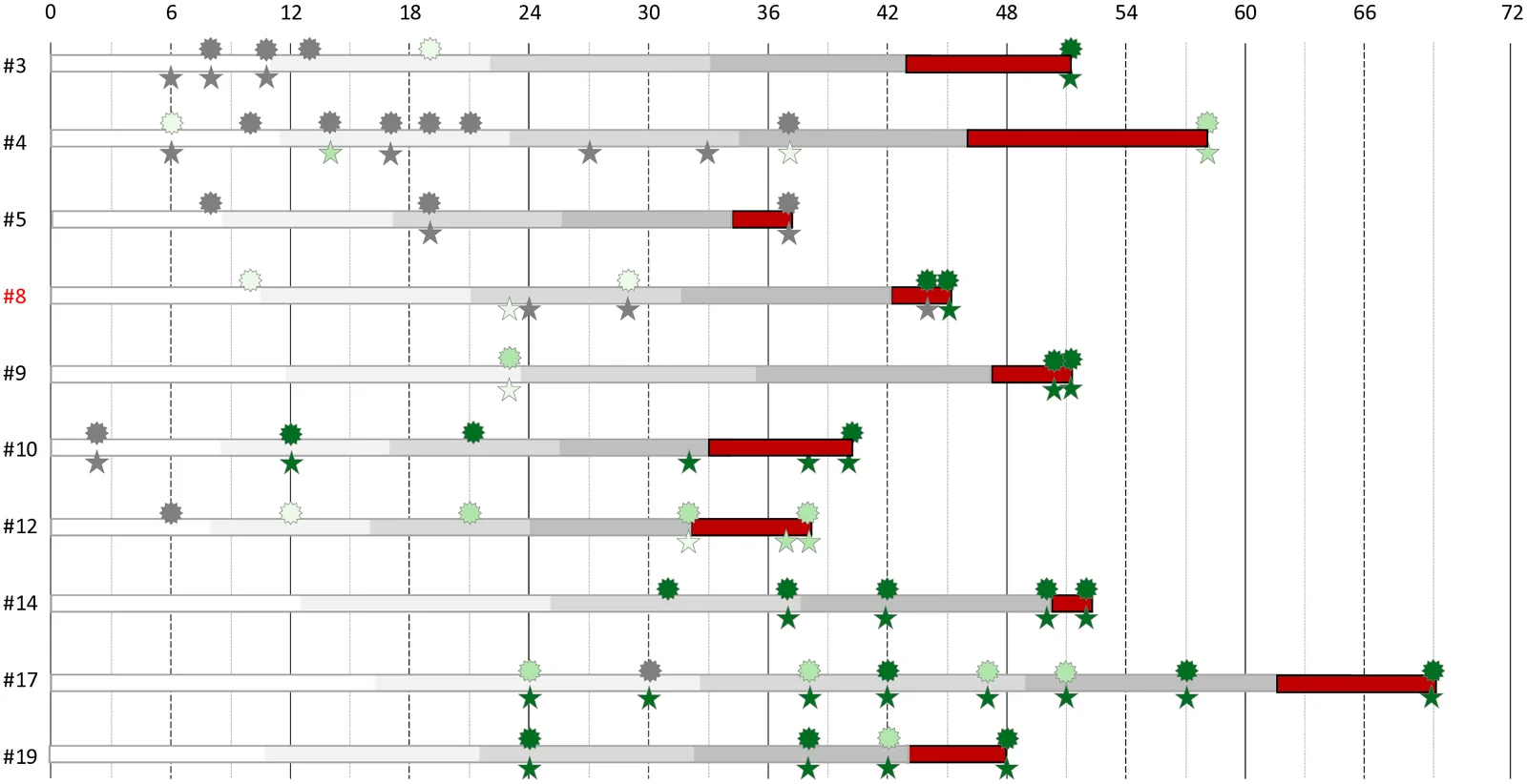
Timeline detection of seeding activity in blood of primates experimentally infected through the intravenous route. Ten primates (ranked as in Figure 2, reference numbers detailed in Table 1) were sampled throughout their silent incubation periods (depicted with a four-levels of grey lane corresponding to the four quartiles) and at the end of their clinical period (depicted with a red lane). They all developed v-CJD but primate #8 developed a myelopathy. For each sample, plasma (12-branch star above the lane) and/or buffy coat (5-branch star under the lane) were analyzed with PMCA. Results are provided as a color code on the basis of duplicates: dark green (strong) = 2/2 positive results after 2 (plasma) or 3 (BC) rounds of PMCA; intermediate green (intermediate) = 2/2 positive results after 3 (plasma) or 4 (BC) rounds of PMCA; dull green (weak) = 2/2 positive or 1/2 low positive results after 4 (plasma) or 5 (BC) rounds of PMCA; gray = 2/2 negative results after 4 (plasma) or 5 (BC) rounds of PMCA. This scoring is summarized in S1 Fig.

### Peripheral prion profiles and false-negative risk

A multivariate analysis was performed to integrate the major indicators of peripheral prion replication, including splenic PrP accumulation, lymphoid involvement, PMCA detection in plasma and buffy coat, and transmission outcomes. Principal component analysis followed by unsupervised k-means clustering identified three major groups of macaques (Fig 3b). Notably, each cluster contained both v-CJD and myelopathic animals, indicating that the clinical phenotype was not the primary determinant of the peripheral replication profile. Instead, animals segregated according to the overall extent of peripheral prion replication. Cluster 1 comprised macaques exhibiting uniformly high levels of peripheral involvement, with positive spleen, extensive lymphoid PrP accumulation, strong PMCA positivity in blood fractions and positive transmission assays (Fig 3c). It is to note that almost all the primates developing v-CJD after transfusion of blood derived samples (#4, #17, #19, #24, except #18) are included in this cluster. Cluster 2 displayed an intermediate phenotype characterized by moderate lymphoid involvement and less consistent positivity across PMCA and transmission assays. Cluster 3 included animals with little or no detectable peripheral PrP replication, which were frequently negative for splenic PrP, PMCA and transmission assays.

To estimate the risk of missing peripheral PrP deposition, we modelled the false-negative rate associated with follicle sampling in v-CJD macaques. False-negative rates decreased sharply with increasing follicle number, with the main gain observed between 1 and 20 follicles (S4 Fig). Region-specific differences became more pronounced at higher follicle counts, with a strong advantage for cervical and submandibular lymph nodes, which showed the lowest false-negative rates when more than 50 follicles were examined, suggesting that they may be preferable targets to reduce the risk of false-negative detection.

### The role of the immune system in peripheral replication

As almost all myelopathic macaques lack apparent detectable levels of abnormal PrP in their lymphoid organs, we aimed to assess the role played by the immune system in their pathophysiology by exposing immunocompetent Swiss and immunodeficient NSG mice IC or IV to samples derived from v-CJD and myelopathic macaques.

After intracerebral inoculation, NSG and Swiss mice exhibited comparable susceptibility to the mouse-adapted BSE strain 6PB1 (181 + /-5 days) [37,38]. Primary transmission of macaque v-CJD through IC route was also complete in both strains (Table 3 and S5 Fig) but with longer incubation periods (305 + /-19 days and 446 + /-20 days in Swiss and NSG mice respectively), and spongiform change was only present in brainstem and spinal cord in NSG mice. After intracerebral inoculation with brain homogenate of myelopathic macaque #8, atypical prion disease phenotypes devoid of apparent PrP^res^ accumulation [30], mainly with a specific spinal involvement (Fig 5), were observed in around 40% of both NSG and Swiss mice. In NSG mice, the incomplete prion disease phenotypes were also observed in 4 mice (13%) exposed to brain samples derived from other myelopathic macaques.

**Table 3:** Disease patterns of transmission in immunocompetent Swiss and immunodeficient NSG mice after IC or IV exposure to primate inocula. For each group, the animals developing v-CJD with detectable cerebral PrP^res^ are counted as PrP^res+^ animals, whereas the animals developing incomplete prion phenotypes without accumulation of cerebral PrP^res^ are counted as PrP^res-^ profiles as previously described [30].

	Immunocompetent Swiss mice				Immunodeficient NSG mice			
Primate inocula	N	PrP^res+^	PrP^res-^	Healthy	N	PrP^res+^	PrP^res-^	Healthy
**Intracerebral**								
v-CJD brain	22	100%	0%	0%	8	100%	0%	0%
Myelopathic #8 brain	10	0%	40%	60%	8	0%	38%	63%
Other myelopathic brains	nd				31	0%	13%	87%
**Intravenous**								
v-CJD brain	20	100%	0%	0%	8	0%	0%	100%
v-CJD blood	222	2%	6%	92%	23	0%	4%	96%
Myelopathic brains					47	0%	4%	96%
Myelopathic blood	143	0%	6%	94%	24	0%	8%	92%
Healthy controls	60	0%	0%	100%	26	0%	0%	100%

**Fig 5:**
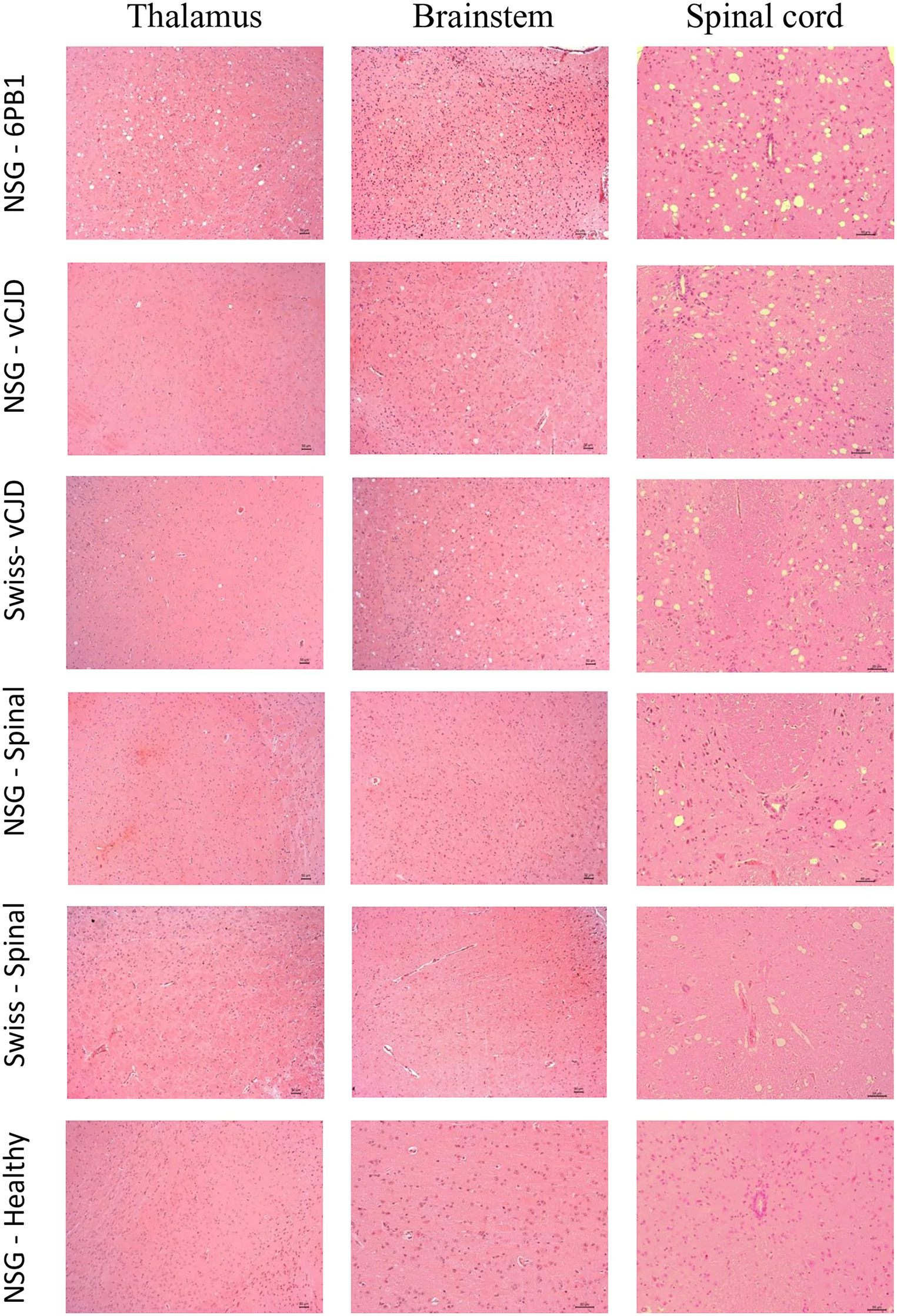
Spongiform changes in immunocompetent and immunodeficient mice. Representative examples of spongiform change observed in NSG or Swiss mice exhibiting a v-CJD or a spinal profile are presented, in parallel to control NSG mice exposed to brain homogenates derived from healthy animals or mice infected with the 6PB1 mouse-adapted BSE strain. Hematoxylin-eosin staining of thalamus (x100), brainstem (x100) and spinal cord (x200).

After intravenous inoculation, the two mouse strains provided different results after exposure to v-CJD-infected brain as expected: transmission of v-CJD occurred in 100% (20/20) of immunocompetent Swiss mice but no recipient NSG immunodeficient mice (0/8) exhibited PrP^res^. Conversely, the same proportions of atypical phenotypes devoid of apparent PrP^res^ accumulation (4–8%) were observed in all the groups of both lineages intravenously exposed to v-CJD or myelopathic blood. No significant differences were observed between the two groups exposed to v-CJD blood (6% vs. 4%; Fisher’s exact test, *p* = 1.000), or between the two groups exposed to myelopathic blood (6% vs. 8%; *p* = 0.660). The overall comparison of the four populations was also non-significant (Fisher–Freeman–Halton exact test, *p* = 0.939). It is to note that atypical phenotypes occur with the same proportion in the NSG mice intravenously exposed to myelopathic brain.

## Discussion

We analyzed here the peripheral distribution of prions in v-CJD experimentally infected macaques, as a key element for the risk assessment of inter-human transmission of v-CJD. BSE contamination of humans mainly occurred through the oral route, presuming a replication of prions targeting the lymphoid organs draining the digestive tract; we previously demonstrated that in macaques the same distribution of abnormal PrP occurs in lymphoid organs after oral or intravenous exposure [24], and here we chose the latter as thought to provide a wider topographical spread of seeding at the exposure, as it has been described in sheep model [39].

Our analyses based on biochemical, immunohistochemical and seeding amplification techniques of different peripheral tissues were mutually consistent: we observed a large heterogeneity of prion replication in lymphoid organs of v-CJD-infected macaques sampled at the terminal stage of the disease, without identifying any obvious correlation with anatomical sites, inocula, or incubation periods. This heterogeneity was observed in macaques exposed to brain products as well as in those exposed to blood products. Such a heterogeneous distribution, unexpected at first sight, has already been described in humans. Indeed, previous reports described v-CJD patients with extremely low or no detectable peripheral PrP^res^ [40,41]. Furthermore, the 2013 UK large-scale study reported heterogeneity in the percentages of positive follicles in appendices of asymptomatic individuals [16], comparable in our primates sampled at the terminal stage of v-CJD (Table 2), although the proportion of highly positive samples in macaques (> 60% positive follicles) was lower, but statistically non-significantly different according to the low numbers of positive human appendices. A preferential targeting of peripheral replication in appendices of orally-exposed individuals may explain this difference.

In addition, 154 (44%) of analyzed LN, including a total of 3366 follicles (mean of 22 ± 19 follicles/LN, ranging from 3 to 138 follicles per lymph node) were found negative in v-CJD-infected primates at the terminal stage of the disease. In sheep, peripheral replication is low at the early stages of incubation period and increases with time [42]. If these observations in both animal models are applicable to humans, the proportion of negative follicles in individuals infected with v-CJD would be higher during the clinically silent incubation period than during the clinical phase (here 44% in macaques); Consequently, prevalence studies based on peripheral organs would include a proportion of false negative individuals that remains to be defined, and this proportion increases as the number of follicles examined decreases: the prevalence of asymptomatic carriers - evaluated at 1/2.000 in the part of the UK population exposed to BSE [16] would thus logically be underestimated. It should be noted here that 10 million people in UK are thought to have been exposed to BSE [43], i.e., more than 100 times the number of people considered to be healthy carriers [16].

To counterbalance this notion that an increase in the prevalence of healthy carriers could improve the risk of secondary iatrogenic transmission, our results suggest that the transfusion risk would be limited to donations from the few donors with the highest levels of peripheral replication: this is consistent with the low number of transfusion-related v-CJD cases reported to date. Indeed, our studies are limited to few samples, but successful transmissions are restricted to those associated with the highest global levels of peripheral replication in lymphoid organs: this is consistent with the concept that lymphocytes get contaminated at the contact of follicular dendritic cells [44]. According to PMCA, v-CJD seeding activity in blood would be a good reflection of overall peripheral replication: blood of macaque #5 devoid of peripheral replication was found negative by PMCA and could not transmit v-CJD, whereas blood samples from macaques #3, #9, and #10 were found highly positive and transmitted v-CJD to mice through intravenous route, and even to macaque through transfusion with blood from macaque #9. This study highlights the heterogeneity of seeding activity in blood from different individuals and subsequently the difficulties of comparing independent studies performed on different samples: Concha-Marambio et al. described their ability to detect seeding activity in the blood of macaques as soon as 2 months after their exposure to v-CJD [45], but those animals were exposed to a much higher dose (the equivalent of 300 mg of infected brain through combination of intraperitoneal and intravenous routes) compared to our studies (maximum of 100 mg intravenously), and thus died from v-CJD in less than 28 months [28]: their lymphoid organs were then massively exposed to contamination and this extreme scenario does not reflect situations observed in humans. However, these results provide further evidence that the PMCA assays used here, which had previously demonstrated high sensitivity on several blood samples collected two years before the onset of clinical signs [20,22,36,46], could help under an industrial format to secure blood-derived products.

For myelopathy, the situation is different. We did not observe with our different approaches any obvious relation between apparent levels of peripheral replication of prion in donor primates and their ability to transmit atypical prion phenotypes. Indeed, seven of ten myelopathic macaques included in this study had no apparent peripheral replication of abnormal PrP, and the three other animals were exposed to inocula that induced v-CJD in replicates with consequent levels of peripheral replication (myelopathic macaques #8, #16 and #20 received inoculum from the same source as v-CJD macaque #9, #14, and #19 respectively). The peripheral abnormal PrP evidenced in these latter three primates might be the signature of parallel replication of the v-CJD strain.

We wondered whether the lack of apparent peripheral replication in lymphoid organs of myelopathic macaques was due to an actual absence of peripheral replication in these organs or to our inability to detect it. We used NSG mice because they combine a non-functional immune system (severe immunodeficiency with an absence of T/ B cells and no functional NK cells [47]) with susceptibilities to both v-CJD and incomplete prion disease-related phenotypes after exposure through IC route that are similar to what we previously described in Swiss mice [30] and observed here. After peripheral exposure, the incidence rates of incomplete prion phenotypes in these mice are similar to those observed in Swiss mice, whereas v-CJD, as expected, was not transmitted [48].

These results suggest that the atypical prion strains linked to incomplete prion phenotypes and macaque myelopathy would not need a functional immune system to replicate before neuroinvasion after peripheral exposure, opposite to v-CJD. Either direct neuroinvasion may occur, as is partially the case in different immunodeficient models [49–51], or peripheral replication would occur elsewhere. This point has not yet been elucidated.

In conclusion, this study revealed that peripheral replication of v-CJD prions is widely heterogeneous and cannot be considered as an ultimate criterion for v-CJD diagnosis, whereas blood-related atypical prion strains rely on different peripheral mechanisms prior to neuroinvasion. At least three epidemiological consequences can be drawn here: (i) the prevalence of silent infected people might be wider than expected when using conventional techniques; (ii) the transfusion v-CJD risk would be limited to the highly infectious samples, which might be detected with current diagnostic techniques; (iii) atypical prions are uncontrollable with current techniques to date and would follow different pathophysiological mechanisms. As deferral measures are lifted in some countries, new tools are needed to analyze and circumvent these newly described novel prion strains.

## Materials and methods

### Ethic statements

Under constant internal veterinary surveillance, animals were housed in accordance with the European Directive 2010/63 related to animal protection and welfare in research. Social enrichment was a constant priority with individual activities and feeding controlled according to the risk of infection. Animals were handled under anaesthesia to limit stress, and euthanasia was performed for ethical reasons when animals lost autonomy. Ethical committee CEtEA N°44 and French Ministry of Research approved the present experiments (approvals 16–034, 17–091 and 20–042). The “reduction” and “refinement” concepts of 3Rs drove the sizes of experimental groups depending on the respective expected rates of transmission. According to the specificity of the model, each macaque is to be considered as a single clinical case.

### Experimental animals

Noveprim (Mauritius) provided captive-bred 2–5-year-old male cynomolgus macaques (*Macaca fascicularis*), all Met/Met homozygous at codon 129 of the prion protein gene (*PRNP),* checked for the absence of common primate pathogens before importation. Janvier Labs (France) provided six weeks-old female Swiss ND4 and NSG (NOD.Cg-Prkdc^scid^Il2rg^tm1Wjl^/SzJ) mice. All the animals were housed in level-3 animal care facilities, randomly allocated to experimental groups and regularly examined at least weekly.

### Intravenous exposure of macaques

Clarified brain homogenates were obtained through successive cycles of sonication-centrifugation as previously described [29], and selected as a representative of the different protocols proposed to model blood infectivity [52]. Blood samples were collected throughout the survival periods of the macaques in citrate anticoagulant and fractionated as previously described [30]. Intravenous exposures were performed by slow injection through saphenous vein under anesthesia (zolazepam/tiletamine 5 mg/kg through intramuscular injection).

For ethical reasons and to ensure comparability among the animals, euthanasia was performed (IV overdose of pentobarbital after anesthesia) when macaques met similar ethical criteria for discontinuation during their clinical phase, that primarily involved a loss of autonomy. In v-CJD macaques, endpoints mostly correspond to cerebellar ataxia, loss of motricity and self-feeding. In myelopathic macaques, endpoints mostly correspond to important paresis/paralysis impairing locomotion or self-feeding. CNS and lymphoid samples (inguinal, popliteal, axillary, submandibular and mesenteric lymph nodes, spleen, tonsils) were sampled and separately coded for biochemical and histological, blindly-performed analyses.

### Bioassay in murine models

Mice were IV or intracerebrally (IC) inoculated with brain homogenates or blood products (frozen-thawed plasma or buffy coat) derived from v-CJD or myelopathic macaques. Negative controls received healthy brain or blood, positive controls received mouse brains infected with the mouse-adapted BSE strain 6PB1 [37,38]. After intraperitoneal anesthesia (ketamine 75 mg/kg + xylazine 10 mg/kg), mice were IC inoculated in the right parietal cortex (20 µl of 1% brain homogenates). IV injections of 200 µl of blood samples or brain homogenates (0,1% wt/vol) were performed in the caudal vein of unanesthetized animals.

Euthanasia by pentobarbital injection (180 mg/kg) was performed when mice exhibited constant signs (motor impairment, weakening, wasting) or at the end of the study. Mice were examined weekly during the preclinical period and daily during the clinical period. The presence of pathological PrP (biochemical and immunohistochemical approaches) and lesions were assessed in their brain and their spinal cord [30]: the disease phenotype was defined according to the resulting observations as previously described [30]. Briefly, a v-CJD profile was characterized by the presence of cerebral PrP^res^. Mice exhibiting clinical signs and spongiform change but devoid of cerebral PrP^res^ were defined as incomplete phenotypes, and subdivided in cerebral, brainstem (bulbar) or spinal profiles depending on the topographical distribution of the lesions.

### Biochemical PrP analysis

PrP was purified according to the TeSeE purification protocol (Bio-Rad) and detected by ELISA (TeSeE kit for primate samples [29], or a specific ELISA for mouse samples using Saf-53 and 11C6 as capture and detection antibodies respectively [53]).

### Histopathology and immunohistochemistry

CNS tissues were fixed in 4% formalin for histological examination. Spongiform change and neuronal lesions were scored on hematoxylin-eosin and Luxol-fast blue stained slides respectively. Peripheral tissues from macaques were fixed in Carnoy’s fluid. Deposits of abnormal PrP were detected using the monoclonal antibody 3F4 after epitope retrieval with guanidinium thiocyanate 4M for 16 minutes (Discovery Automated IHC Stainer, Ventana). For each lymph node, the number of positive and total lymphoid follicles were scored (110–1713 follicles were observed per animal).

### Protein misfolding cyclic amplification (PMCA)

Two different techniques were performed as previously described for PMCA of abnormal PrP, on two independent blind series of buffy coat [20] and plasma samples [22] issued from the same blood collection respectively, under the conditions listed in S4 Table.

Briefly, PMCA amplification of buffy coat samples (5 microliters) was performed as previously described [20] using brains from transgenic mice that express ovine A_136_R_154_Q_171_ PrP variant PrP^c^ (tgShXI) as PMCA substrate [46,54]. Conversely, PMCA on plasma samples (450 microliters) was performed after an initial step of capture on plasminogen-coated beads as previously described [22], using brain homogenate from healthy humanized transgenic mice overexpression the Met129 PrP (tg650) as PMCA substrate [55].

Each PMCA run included unseeded controls and serial ten-fold dilutions of a reference v-CJD sample (10% brain homogenate) as controls for the amplification efficiency. Brain, spinal cord and spleen were also analysed by PMCA for several animals after serial ten-fold dilutions. After each round, reaction products (1 volume) were mixed with the fresh substrate (9 volumes) to seed the following round while a part of the same product was analysed by Western Blot for the presence of PK-resistant PrP. A semi-quantitative scoring was attempted based on the first round which turned out to be positive (details in S1 Fig).

### Statistical analysis of PrP positivity patchiness and effect on phenotype

Follicular PrP positivity was analysed using generalized linear mixed models applied to count-derived proportions. At the lymph-node level, the number of PrP-positive follicles out of the total number of follicles examined for a lymph node in a primate was modelled using a beta-binomial distribution to account for overdispersion. Inter-individual heterogeneity was quantified using the intraclass correlation coefficient (ICC). The significance of the random effect was assessed by likelihood-ratio comparison between models with and without the random intercept. The effect of lymph-node anatomy was tested by including region as a fixed effect. Statistical significance was assessed using analysis of deviance with F tests.

Correlations among peripheral prion replication markers were assessed using Spearman's rank correlation. Principal component analysis (PCA) was performed on standardized variables, followed by k-means clustering to identify groups of macaques with similar peripheral prion replication profiles.

### Modelling false-negative detection probability

To estimate how lymph-node region and follicle-sampling depth influence the risk of false-negative detection of peripheral PrP deposition, we modelled the probability of failing to detect any positive follicle in v-CJD macaques when a fixed number of follicles was sampled from a given anatomical lymph-node region. Only macaques classified as v-CJD were included. Replicate lymph nodes from the same anatomical region were merged within each animal, and right- and left-sided samples were pooled to define a single anatomical region. For each animal-region pair, the probability of a false-negative result was calculated using the exact hypergeometric probability of drawing no positive follicles when sampling k follicles without replacement from the observed number of positive and total follicles. Sampling depths of 1, 5, 10, 20, 30, 50, and 100 follicles were evaluated independently for each lymph-node region. Region-level false-negative rates and empirical 95% intervals were then estimated by 10,000 bootstrap resamplings of animals within each lymph-node region.

## Supporting information

S1 Fig Examples of PMCA results and scoring. Two distinct techniques of PMCA were used for detection of PrP seeding activity in plasma or buffy coat, using a) (plasminogen-coated beads) capture or b) direct techniques respectively. For each technique, the sensitivity of detection in infected brain and representative examples of detection of amplified PrP^res^ in blood samples are provided. For these samples, the reference of macaque and the time of sampling (expressed as a % of their survival period) are specified. c) resumes the quotation of PMCA positivity for blood samples depending on the numbers of round to be achieved for turning positive.
(TIF)

S2 Fig Distribution of prion positivity in lymph nodes examined for each macaque. For the 18 macaques exhibiting positivity in at least one lymph node (all except BSE/v-CJD #5 primate and myelopathic #15, #16, #21, #22, #23, #25, #26 and #27 macaques), each analyzed lymph node is mentioned at its anatomical site with a color code depending on the % of follicles detected as positive.
(TIF)

S3 Fig Transmission experiments through transfusion of pools of blood of primates experimentally infected through intravenous routes. Five (#3, #4, #5, #6 and #7) or six (#8, #9, #10, #11, #12 and #13) donor primates were identically inoculated through intravenous routes with brains from BSE or v-CJD infected macaques respectively (details of inoculation have been already published [29]). Their blood was sampled during their clinical stage, pooled and transfused to recipient macaques #14 and #17 respectively. Red blood cells from these pools were fractionated and transfused before (#19 and #20) or after (#15, #16 and #18) passage through a leukoreduction filter. For all the macaques, the disease issue is specified (brown animal: v-CJD; yellow animal: myelopathy). Their brain, spinal cord and spleen have been tested for the presence of PrP^res^ by ELISA, evidenced here through the color of corresponding square (red: high accumulation (OD > 1.500) – pink = low accumulation (limit of detection < OD < 1.500) – grey = negative (OD < limit of detection)) and also for some of them was tested seeding activity by PMCA, whose last positive dilution is mentioned within the corresponding right square (if only one square is present, PMCA was not performed). The positivity of their lymphoid organs and blood components is mentioned according to the same rules as described in Fig 2.
(TIF)

S4 Fig Estimated false-negative rate for peripheral PrP detection according to lymph-node region and follicle sampling depth. Boxplots show the distribution of false-negative rates obtained from 10,000 bootstrap simulations for each anatomical lymph-node region and sampling depth. The false-negative probability for each animal was calculated using the exact hypergeometric distribution based on the observed numbers of positive and total follicles. Horizontal dashed lines indicate false-negative rate thresholds of 1%, 5%, and 10%.
(TIF)

S5 Fig Survival periods of Swiss and NSG mice after exposure to brain and blood products derived from CJD and myelopathic macaques. Swiss (a) and NSG (b) mice were exposed through intracerebral or intravenous routes to brain or blood samples derived from v-CJD or myelopathic macaques. Box plots are presented for each group, and survival period of each animal is symbolized by a circle (white: no disease; green: PrP^res+^ v-CJD profile; red: PrP^res-^ incomplete phenotype profile).
(TIF)

S6 Raw Images Raw western blots presented in S1 Fig. (PDF)

S1 Table Detailed distribution of PrP accumulation in individual lymph nodes of the 27 macaques included in this study identified by PrP immunohistochemistry. For each lymph node (LN), defined by its anatomical position (tonsils, cervical, submandibular, axillary, inguinal, popliteal, mesenteric or retroperitoneal lymph nodes. L for Left and R for Right), the total number of follicles, and among them the positively stained ones, were counted. For each anatomical position, several lymph nodes were harvested (numbers can vary from one animal to another).
(PDF)

S2 Table Detailed disease transmissions to Swiss mice through transfusion of primate-derived blood products. Blood of nine primates (six v-CJD and 3 myelopathic animals) was sampled at the terminal stage of their disease and fractionated under centrifugation. Each frozen/thawed plasma (200 ml) or buffy coat (20 ml) sample was injected to 18–39 mice through intravenous or intracerebral route respectively, both of them being equivalent to 400 ml of whole blood. In parallel, seeding activity in these samples was assessed by PMCA reaction (The results of PMCA analysis are provided as a qualitative scoring summarized in S1 Fig). Among all the corresponding recipient mice, those exhibiting clinical signs are reported according to the presence of cerebral PrP^res^ as classical, PrP^res^ positive v-CJD profile, or atypical, PrP^res^ negative incomplete prion profiles. For these latter ones, the animals were sub-divided according to their lesional profile (focused on brainstem, brain or spinal cord according to the incomplete phenotype profiles previously described in 30) when assessed (lesional profile was not performed for 3 mice respectively exposed to plasma from primate #3, buffy coat from primate #9 and plasma from primate #14). No lesion was observed in animals devoid of clinical sign.
(DOCX)

S3 Table Detection of abnormal PrP seeding activity in plasma or buffy coat samples from infected macaques 1–27, and 12 healthy control macaques (noted from A to L). The time of sampling is indicated relative to the incubation period, the onset of clinical signs and as a proportion of the survival period. All the durations are expressed as months. Plasma samples have been tested in duplicates, and some of them in quadruplicates; buffy coat samples were tested in quadruplicates. (nd: not done since previous rounds are full positive).
(DOCX)

S4 Table Parameters and characteristics of PMCA techniques. (DOCX)

## Abbreviations

BSE: bovine spongiform encephalopathy
CNS: central nervous system
IC: intracerebral
IHC: immunohistochemistry
IV: intravenous
LN: lymph nodes
NSG: non-obese diabetic SCID gamma
PrP: prion protein
RBC: red blood cell
UK: United Kingdom
v-CJD: variant Creutzfeldt-Jakob disease
