# Supplementary material for: Heterogeneous transfusion risk linked to variant Creutzfeldt-Jakob disease in a non-human primate model
Source: PLoS Pathog. 2026 Nov 10;22(11):e1014769. doi: 10.1371/journal.ppat.1014769 (PMC13766618; doi:10.1371/journal.ppat.1014769)

CAPTURE PMCA (PLASMA)

Serial dilutions

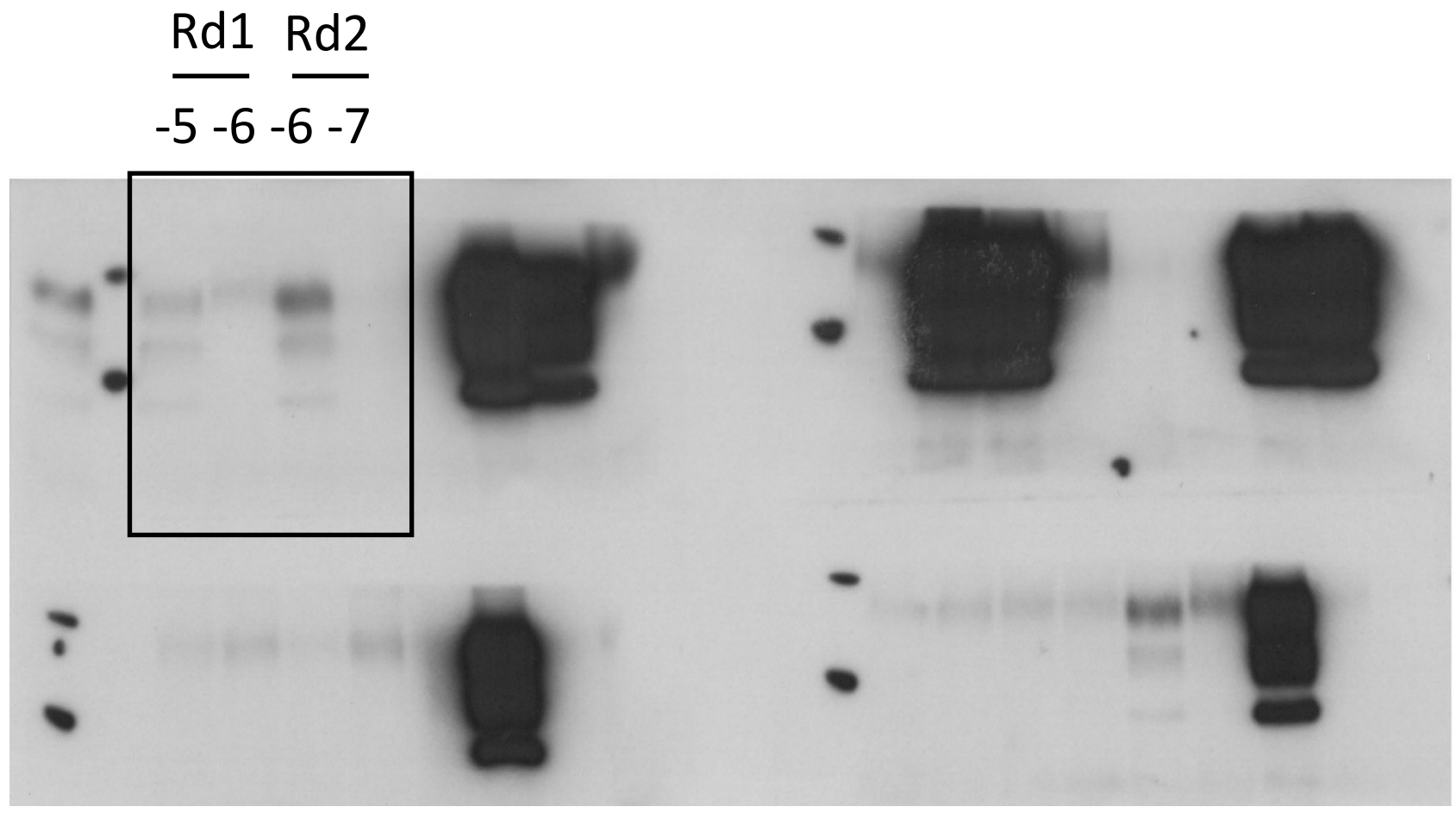

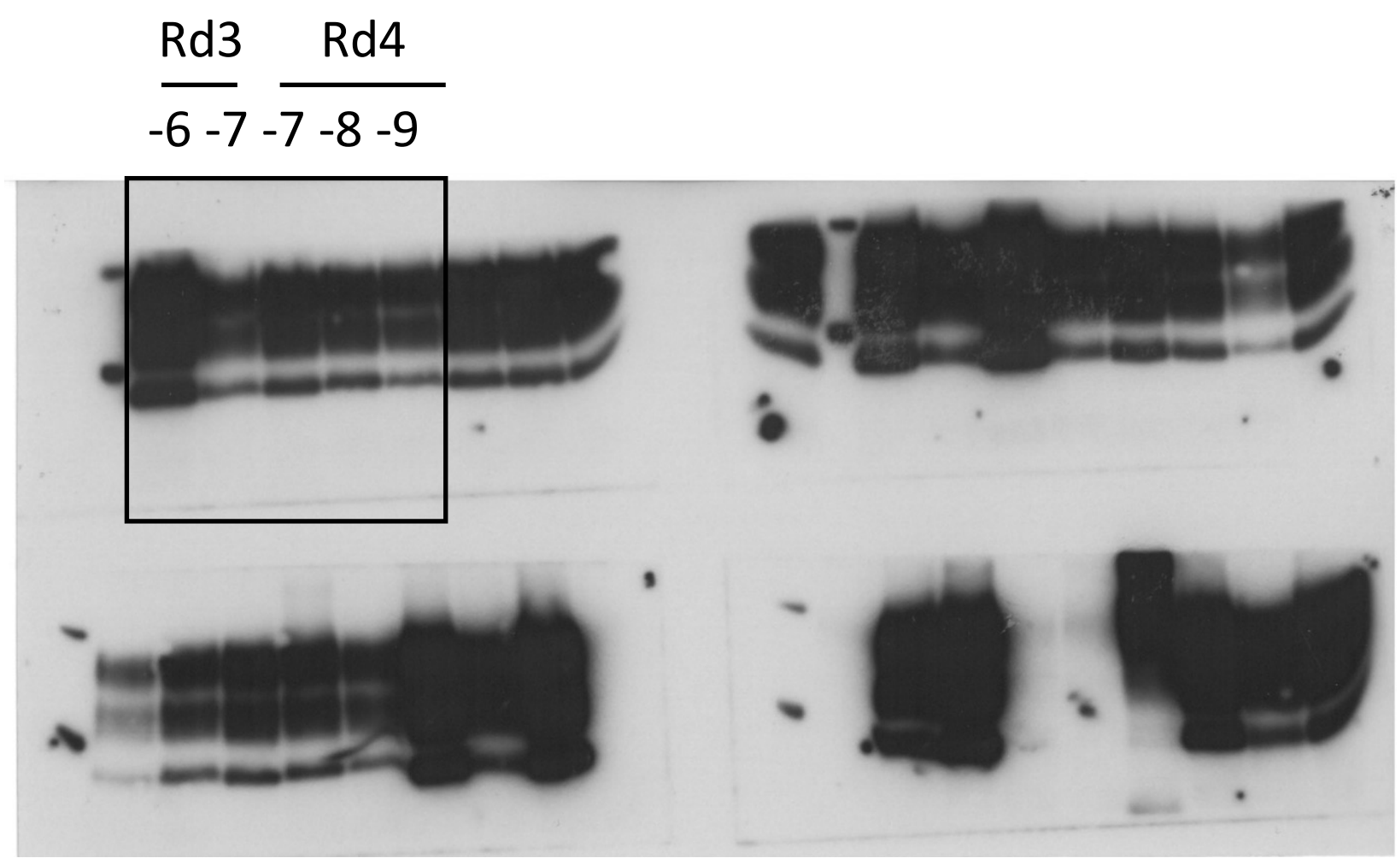

Samples

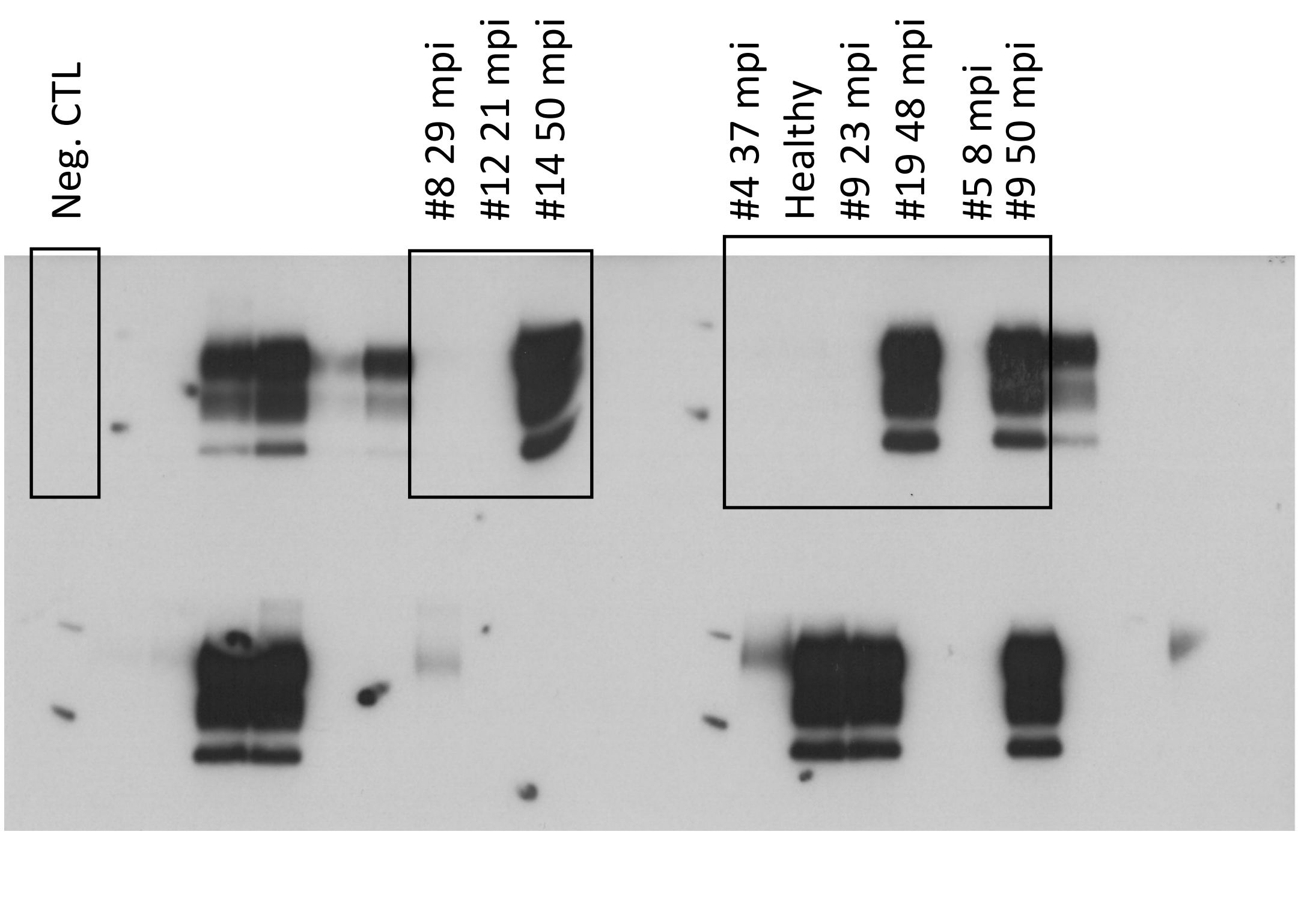

DIRECT PMCA (buffy coat)

Serial dilutions / Round 1

PUBLI – gel N°1

Migration sur mini gel 12% - semi dry

Ac Iaire : SHA 31 ascite au 1/8000ème

Ac IIaire: Goat anti-mouse/HRP au 1/5000ème

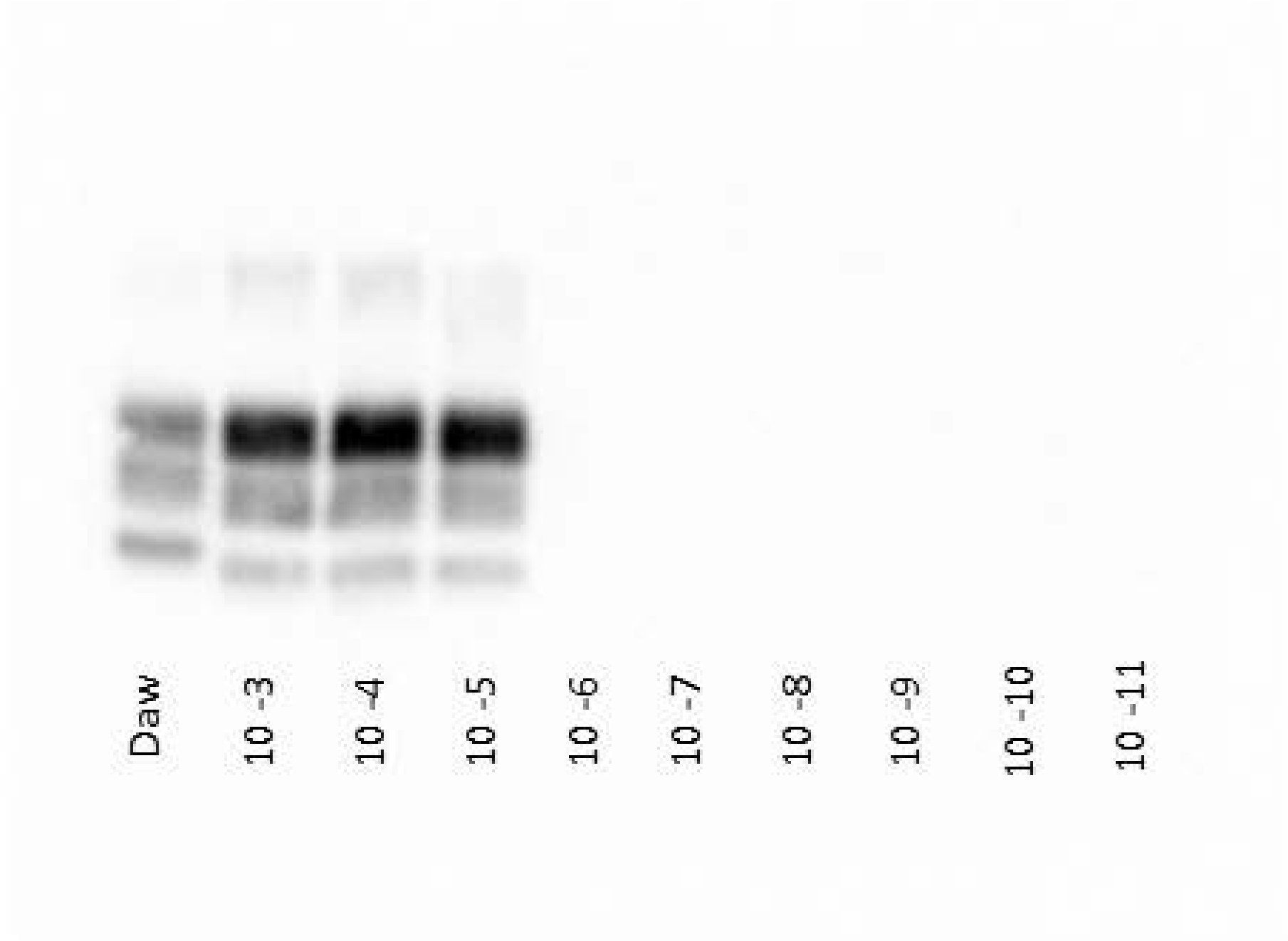

DIRECT PMCA (buffy coat)

Serial dilutions / Round 2

PUBLI – gel N°2

Migration sur mini gel 12% - semi dry

Ac Iaire : SHA 31 ascite au 1/8000ème

Ac IIaire: Goat anti-mouse/HRP au 1/5000ème

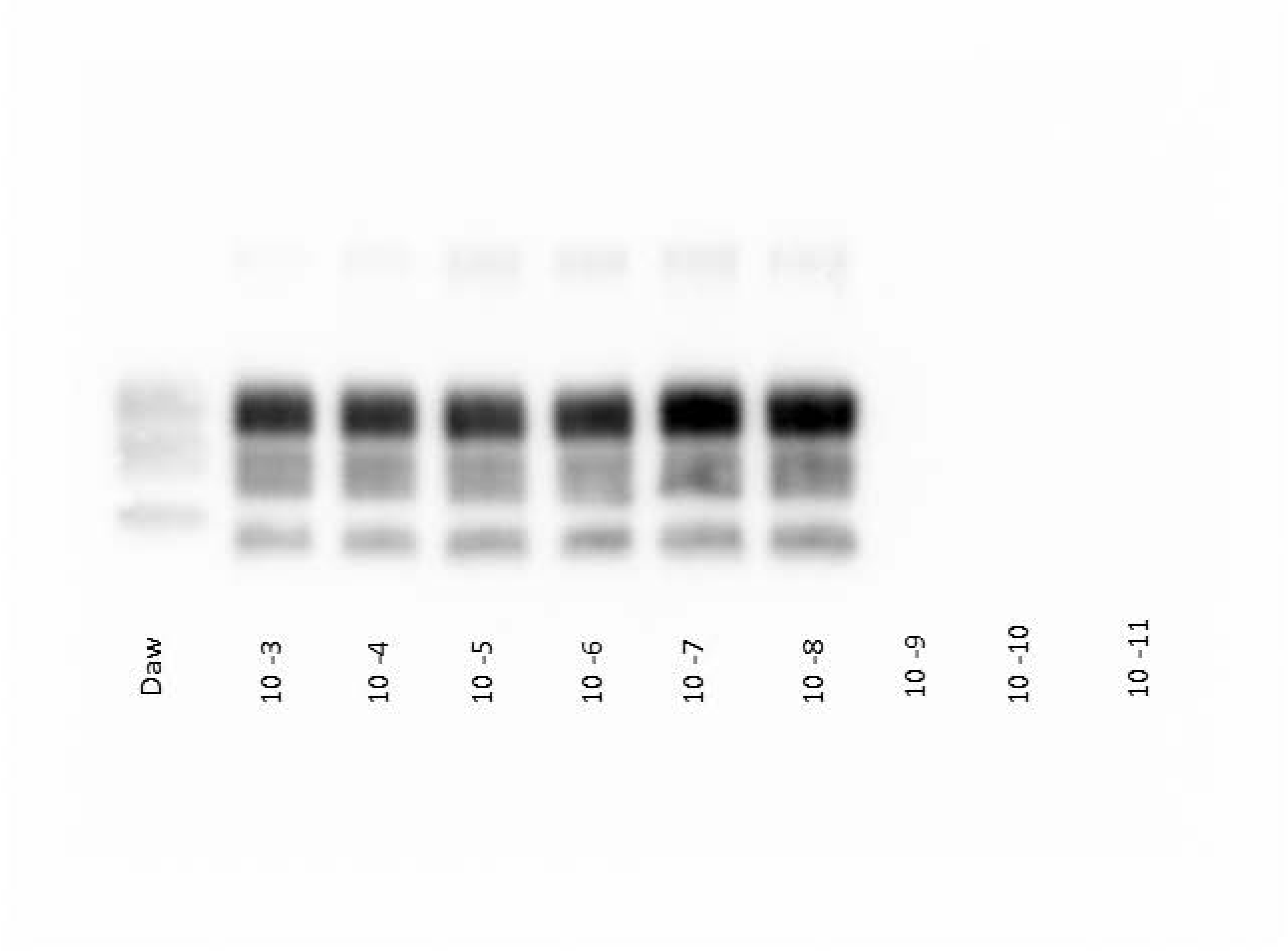

DIRECT PMCA (buffy coat)

Serial dilutions / Round 3

PUBLI – gel N°3

Migration sur mini gel 12% - semi dry

Ac Iaire : SHA 31 ascite au 1/8000ème

Ac IIaire: Goat anti-mouse/HRP au 1/5000ème

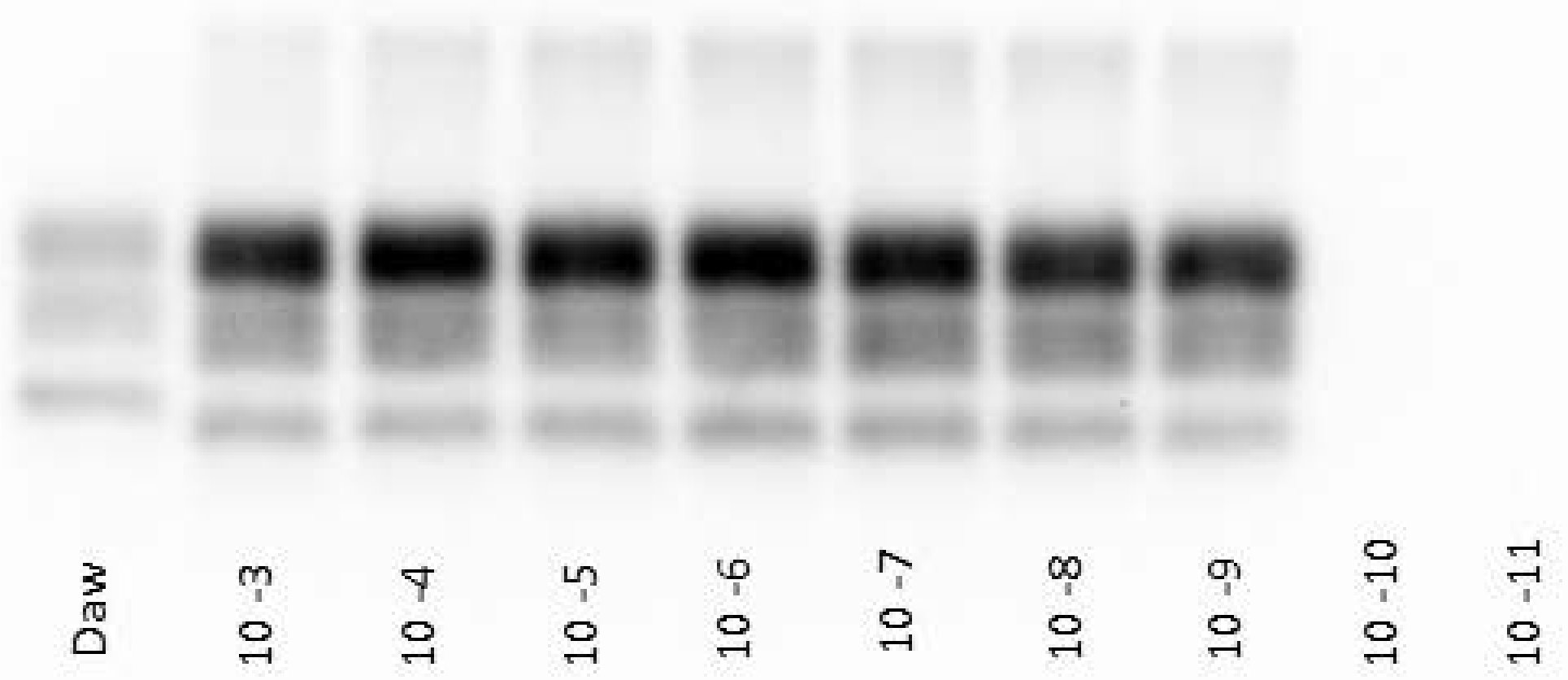

DIRECT PMCA (buffy coat)

Samples 1/2

PUBLI – gel N°4

Migration sur mini gel 12% - semi dry

Ac Iaire : SHA 31 ascite au 1/8000ème

Ac IIaire: Goat anti-mouse/HRP au 1/5000ème

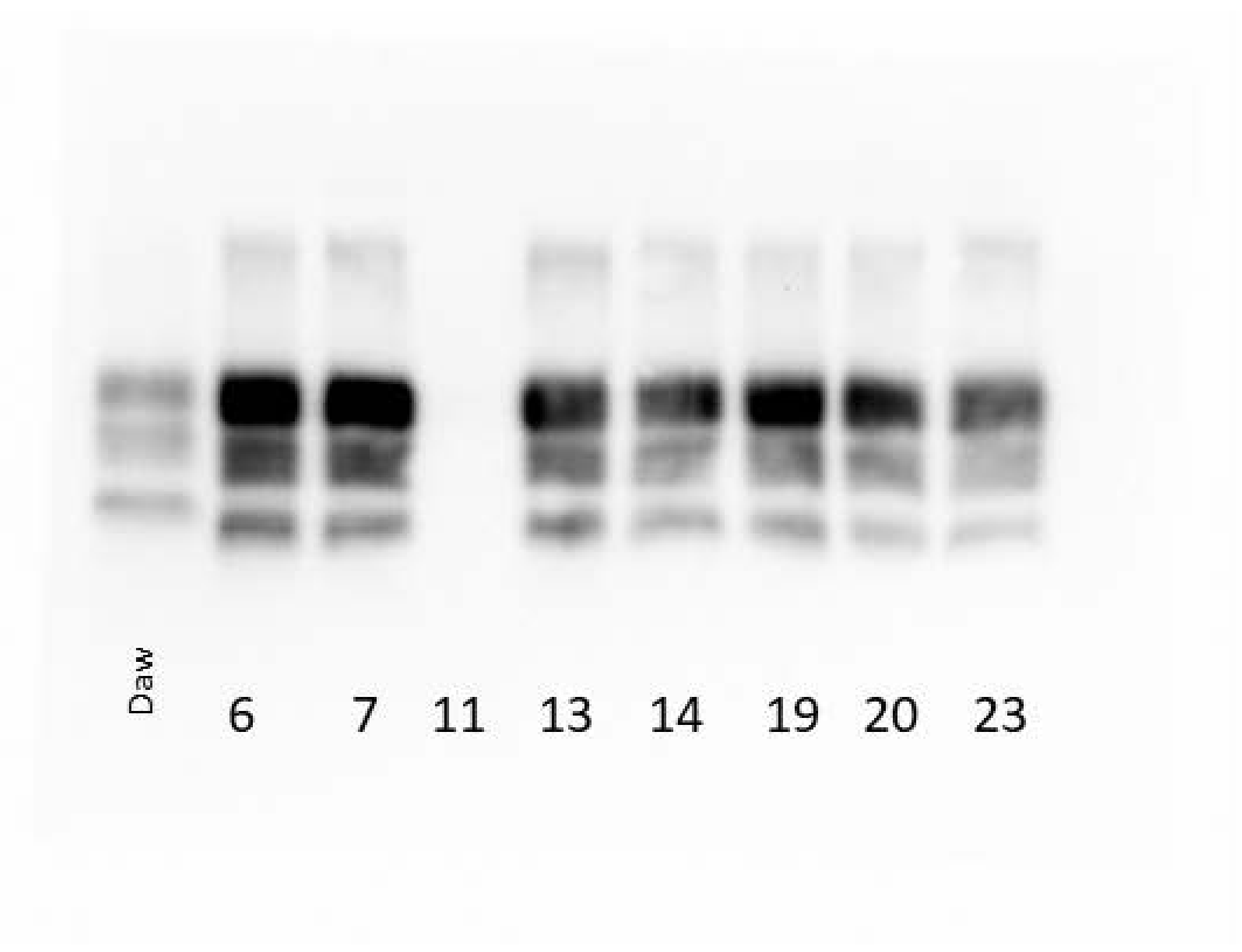

DIRECT PMCA (buffy coat)

Samples 2/2

PUBLI – gel N°5

Migration sur mini gel 12% - semi dry

Ac Iaire : SHA 31 ascite au 1/8000ème

Ac IIaire: Goat anti-mouse/HRP au 1/5000ème

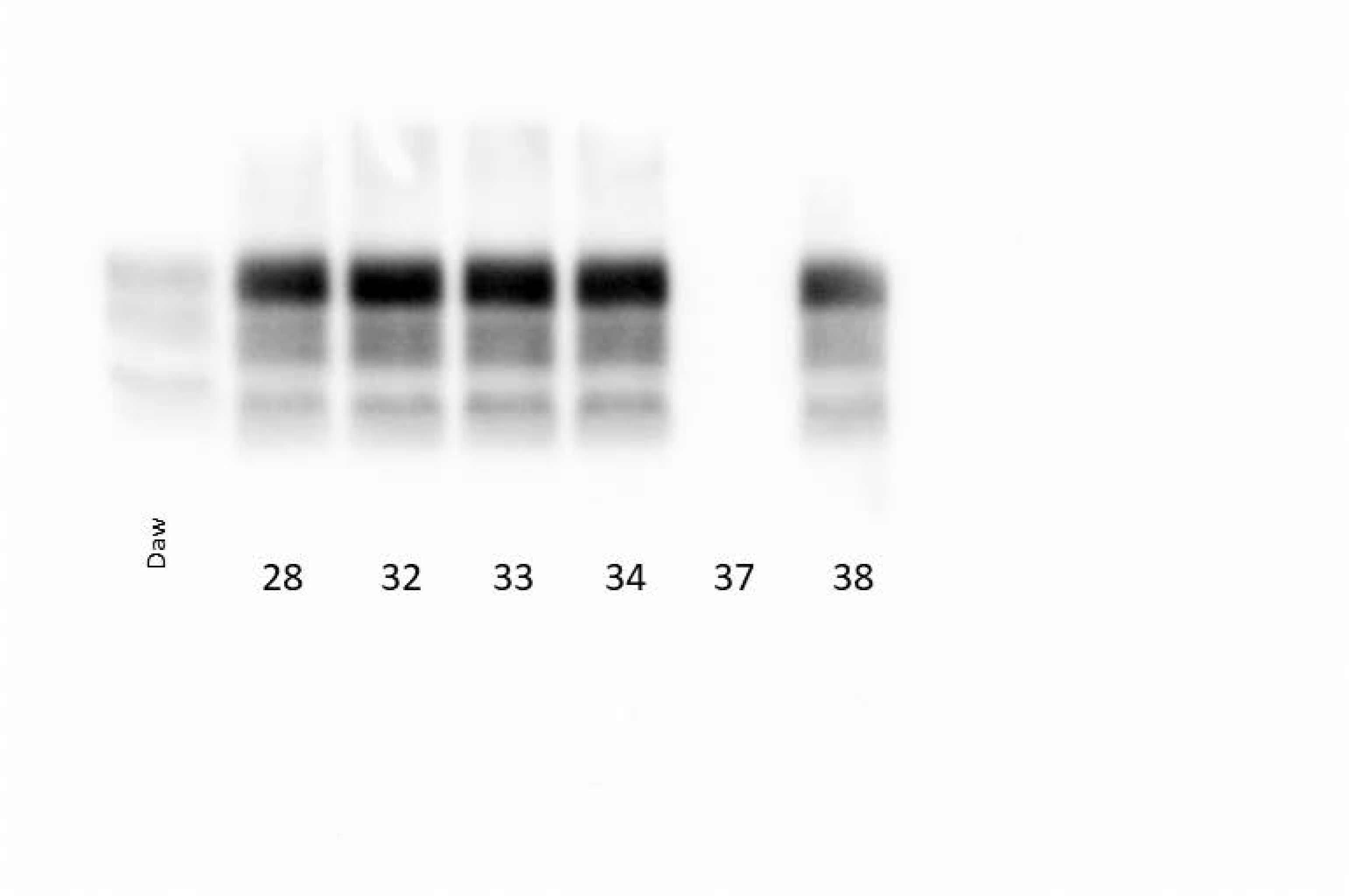

Supplement: Supplementary file 1 [file ppat.1014769.s006.pdf]
