# Supplementary material for: Heterogeneous transfusion risk linked to variant Creutzfeldt-Jakob disease in a non-human primate model
Source: PLoS Pathog. 2026 Nov 10;22(11):e1014769. doi: 10.1371/journal.ppat.1014769 (PMC13766618; doi:10.1371/journal.ppat.1014769)
Supplement: Supplementary file 2 [file ppat.1014769.s007.pdf]

Primate Identity	#1			#2			#3			#4			#5			#6		
	Positive follicles	Total follicles	% positive	Positive follicles	Total follicles	% positive	Positive follicles	Total follicles	% positive	Positive follicles	Total follicles	% positive	Positive follicles	Total follicles	% positive	Positive follicles	Total follicles	% positive
Tonsil #1										0	12	0%	0	5	0%	5	28	18%
Tonsil #2										0	8	0%				9	13	69%
Tonsil #3																		
Tonsil #4																		
Tonsil #5																		
Tonsil #6																		
Tonsil #7																		
Tonsil #8																		
Cervical LN #1							11	25	44%									
Cervical LN #2																		
Cervical LN #3																		
Cervical LN #4																		
Cervical LN #5																		
Cervical LN #6																		
Submandibular LN R #1				32	44	73%	0	15	0%	4	24	17%						
Submandibular LN R #2				8	16	50%	17	78	22%									
Submandibular LN R #3							10	40	25%									
Submandibular LN R #4																		
Submandibular LN L #1				28	46	61%	8	46	17%							20	41	49%
Submandibular LN L #2																19	47	40%
Submandibular LN L #3																19	42	45%
Submandibular LN L #4																		
Axillary LN R #1	0	41	0%	5	24	21%	2	22	9%	0	40	0%				1	40	3%
Axillary LN R #2				0	11	0%										1	40	3%
Axillary LN R #3																1	28	4%
Axillary LN R #4																		
Axillary LN R #5																		
Axillary LN L #1							25	50	50%	0	40	0%	0	40	0%	0	99	0%
Axillary LN L #2							12	39	31%									
Axillary LN L #3																		
Axillary LN L #4																		
Axillary LN L #5																		
Axillary LN L #6																		
Axillary LN L #7																		
Inguinal LN R #1							3	17	18%	0	8	0%	0	18	0%	0	46	0%
Inguinal LN R #2																0	21	0%
Inguinal LN R #3																0	24	0%
Inguinal LN R #4																		
Inguinal LN R #5																		
Inguinal LN R #6																		
Inguinal LN L #1							19	56	34%	0	10	0%	0	44	0%	0	48	0%
Inguinal LN L #2										0	21	0%				0	22	0%
Inguinal LN L #3																0	21	0%
Inguinal LN L #4																		
Inguinal LN L #5																		
Inguinal LN L #6																		
Inguinal LN L #7																		
Inguinal LN L #8																		
Inguinal LN L #9																		
Inguinal LN L #10																		
Inguinal LN L #11																		
Popliteal LN R#1							2	20	10%							1	14	7%
Popliteal LN R#2																1	10	10%
Polpiteal LN L #1							6	6	100%							0	15	0%
Polpiteal LN L #2																0	13	0%
Mesenteric LN #1	50	69	72%				3	14	21%	6	10	60%	0	115	0%	2	30	7%
Mesenteric LN #2										1	8	13%						
Mesenteric LN #3										1	51	2%						
Mesenteric LN #4										4	12	33%						
Mesenteric LN #5										0	21	0%						
Mesenteric LN #6																		
Mesenteric LN #7																		
Mesenteric LN #8																		
Mesenteric LN #9																		
Mesenteric LN #10																		
Mesenteric LN #11																		
Mesenteric LN #12																		
Mesenteric LN #13																		
Mesenteric LN #14																		
Mesenteric LN #15																		
Mesenteric LN #16																		
Mesenteric LN #17																		
Mesenteric LN #18																		
Mesenteric LN #19																		
Mesenteric LN #20																		
Mesenteric LN #21																		
Mesenteric LN #22																		
Mesenteric LN #23																		
Mesenteric LN #24																		
Mesenteric LN #25																		
Mesenteric LN #26																		
Mesenteric LN #27																		
Mesenteric LN #28																		
Mesenteric LN #29																		
Mesenteric LN #30																		
Mesenteric LN #31																		
Retroperitoneal LN #1							4	20	20%	0	15	0%						
Retroperitoneal LN #2							60	126	48%									
Retroperitoneal LN #3																		
Retroperitoneal LN #4																		
Retroperitoneal LN #5																		
**Total follicles**	**50**	**110**	**45%**	**73**	**141**	**52%**	**182**	**574**	**32%**	**16**	**280**	**6%**	**0**	**222**	**0%**	**79**	**642**	**12%**
			**0-72%**			**0_21-73%**			**0_9-50%**			**0_2-60%**						**0_3-69%**
	**Positive**	**Total**		**Positive**	**Total**		**Positive**	**Total**		**Positive**	**Total**		**Positive**	**Total**		**Positive**	**Total**	
**Lymph Nodes**	**1**	**2**	**50%**	**4**	**5**	**80%**	**14**	**15**	**93%**	**5**	**14**	**36%**	**0**	**5**	**0%**	**11**	**20**	**55%**
**Spleen (O.D.) Cut-off = 0.100**			**0.503**			**2.139**			**2.930**			**0.297**			**0.040**			**0.167**

Primate Identity	#7			#8			#9			#10			#11			#12		
	Positive follicles	Total follicles	% positive	Positive follicles	Total follicles	% positive	Positive follicles	Total follicles	% positive	Positive follicles	Total follicles	% positive	Positive follicles	Total follicles	% positive	Positive follicles	Total follicles	% positive
**Tonsil #1**	0	7	**0%**	7	36	**19%**	6	18	**33%**				7	21	**33%**			
**Tonsil #2**													19	25	**76%**			
**Tonsil #3**																		
**Tonsil #4**																		
**Tonsil #5**																		
**Tonsil #6**																		
**Tonsil #7**																		
**Tonsil #8**																		
**Cervical LN #1**													7	20	**35%**	1	15	**7%**
**Cervical LN #2**													15	28	**54%**			
**Cervical LN #3**													4	9	**44%**			
**Cervical LN #4**																		
**Cervical LN #5**																		
**Cervical LN #6**																		
**Submandibular LN R #1**	16	76	**21%**				3	110	**3%**	17	36	**47%**	30	66	**45%**	7	17	**41%**
**Submandibular LN R #2**										12	50	**24%**						
**Submandibular LN R #3**										3	13	**23%**						
**Submandibular LN R #4**										20	36	**56%**						
**Submandibular LN L #1**							12	150	**8%**	2	26	**8%**	18	35	**51%**	30	45	**67%**
**Submandibular LN L #2**										3	26	**12%**						
**Submandibular LN L #3**										24	86	**28%**						
**Submandibular LN L #4**										9	20	**45%**						
**Axillary LN R #1**	0	36	**0%**				9	102	**9%**	5	22	**23%**	8	23	**35%**			
**Axillary LN R #2**										5	25	**20%**	16	36	**44%**			
**Axillary LN R #3**										10	40	**25%**	18	26	**69%**			
**Axillary LN R #4**										20	40	**50%**	3	11	**27%**			
**Axillary LN R #5**													0	6	**0%**			
**Axillary LN L #1**	7	64	**11%**				2	68	**3%**				0	12	**0%**			
**Axillary LN L #2**													0	19	**0%**			
**Axillary LN L #3**													0	20	**0%**			
**Axillary LN L #4**													7	25	**28%**			
**Axillary LN L #5**																		
**Axillary LN L #6**																		
**Axillary LN L #7**																		
**Inguinal LN R #1**	8	56	**14%**	0	49	**0%**	34	68	**50%**				4	10	**40%**			
**Inguinal LN R #2**													10	27	**37%**			
**Inguinal LN R #3**													0	17	**0%**			
**Inguinal LN R #4**																		
**Inguinal LN R #5**																		
**Inguinal LN R #6**																		
**Inguinal LN L #1**	0	50	**0%**	0	26	**0%**	5	85	**6%**				7	73	**10%**			
**Inguinal LN L #2**													3	17	**18%**			
**Inguinal LN L #3**													5	31	**16%**			
**Inguinal LN L #4**													11	40	**28%**			
**Inguinal LN L #5**													0	9	**0%**			
**Inguinal LN L #6**													0	16	**0%**			
**Inguinal LN L #7**																		
**Inguinal LN L #8**																		
**Inguinal LN L #9**																		
**Inguinal LN L #10**																		
**Inguinal LN L #11**																		
**Popliteal LN R#1**										5	19	**26%**						
**Popliteal LN R#2**																		
**Polpiteal LN L #1**							0	17	**0%**	3	24	**13%**						
**Polpiteal LN L #2**							0	60	**0%**									
**Mesenteric LN #1**	0	18	**0%**							5	6	**83%**	15	26	**58%**	0	110	**0%**
**Mesenteric LN #2**	0	20	**0%**							11	44	**25%**	12	22	**55%**	1	50	**2%**
**Mesenteric LN #3**										6	56	**11%**	5	8	**63%**	2	14	**14%**
**Mesenteric LN #4**										9	54	**17%**				3	19	**16%**
**Mesenteric LN #5**																2	7	**29%**
**Mesenteric LN #6**																		
**Mesenteric LN #7**																		
**Mesenteric LN #8**																		
**Mesenteric LN #9**																		
**Mesenteric LN #10**																		
**Mesenteric LN #11**																		
**Mesenteric LN #12**																		
**Mesenteric LN #13**																		
**Mesenteric LN #14**																		
**Mesenteric LN #15**																		
**Mesenteric LN #16**																		
**Mesenteric LN #17**																		
**Mesenteric LN #18**																		
**Mesenteric LN #19**																		
**Mesenteric LN #20**																		
**Mesenteric LN #21**																		
**Mesenteric LN #22**																		
**Mesenteric LN #23**																		
**Mesenteric LN #24**																		
**Mesenteric LN #25**																		
**Mesenteric LN #26**																		
**Mesenteric LN #27**																		
**Mesenteric LN #28**																		
**Mesenteric LN #29**																		
**Mesenteric LN #30**																		
**Mesenteric LN #31**																		
**Retroperitoneal LN #1**													0	20	**0%**	0	10	**0%**
**Retroperitoneal LN #2**													0	16	**0%**	0	6	**0%**
**Retroperitoneal LN #3**													0	6	**0%**	0	12	**0%**
**Retroperitoneal LN #4**																0	18	**0%**
**Retroperitoneal LN #5**																3	18	**17%**
**Total follicles**	**31**	**327**	**9%**	**7**	**111**	**6%**	**71**	**678**	**10%**	**169**	**623**	**27%**	**224**	**720**	**31%**	**49**	**341**	**14%**
			**0_11-21%**			**0_19%**			**0_3-50%**			**8-83%**			**0_10-76%**			**0_2-67%**
	**Positive**	**Total**		**Positive**	**Total**		**Positive**	**Total**		**Positive**	**Total**		**Positive**	**Total**		**Positive**	**Total**	
**Lymph Nodes**	**3**	**8**	**38%**	**1**	**3**	**33%**	**7**	**9**	**78%**	**18**	**18**	**100%**	**21**	**31**	**68%**	**8**	**13**	**62%**
**Spleen (O.D.) Cut-off = 0.100**			**0.040**			**0.390**			**0.270**			**2.925**			**0.140**			**0.100**

Primate Identity	#13			#14			#15			#16			#17			#18		
	Positive follicles	Total follicles	% positive	Positive follicles	Total follicles	% positive	Positive follicles	Total follicles	% positive	Positive follicles	Total follicles	% positive	Positive follicles	Total follicles	% positive	Positive follicles	Total follicles	% positive
Tonsil #1	0	8	0%	40	80	50%				0	14	0%				1	47	2%
Tonsil #2	0	7	0%	40	112	36%				0	20	0%				1	29	3%
Tonsil #3	0	15	0%	36	108	33%										4	20	20%
Tonsil #4	0	33	0%	8	50	16%												
Tonsil #5	12	22	55%															
Tonsil #6	0	25	0%															
Tonsil #7	6	15	40%															
Tonsil #8																		
Cervical LN #1																0	18	0%
Cervical LN #2																0	15	0%
Cervical LN #3																1	14	7%
Cervical LN #4																0	24	0%
Cervical LN #5																		
Cervical LN #6																		
Submandibular LN R #1	0	26	0%	0	36	0%							6	16	38%	9	44	20%
Submandibular LN R #2	0	28	0%	16	32	50%							5	14	36%	0	138	0%
Submandibular LN R #3	0	50	0%	16	63	25%							7	9	78%	1	44	2%
Submandibular LN R #4																		
Submandibular LN L #1	2	60	3%	0	27	0%				0	19	0%	8	15	53%	0	19	0%
Submandibular LN L #2				1	32	3%							10	27	37%	0	18	0%
Submandibular LN L #3				15	84	18%							10	23	43%	6	50	12%
Submandibular LN L #4													9	30	30%	1	37	3%
Axillary LN R #1				0	30	0%	0	28	0%	0	19	0%	1	21	5%	0	18	0%
Axillary LN R #2				5	86	6%	0	25	0%	0	36	0%	10	23	43%	0	15	0%
Axillary LN R #3										0	15	0%				0	10	0%
Axillary LN R #4																0	12	0%
Axillary LN R #5																0	28	0%
Axillary LN L #1							0	18	0%	0	16	0%	5	30	17%	0	19	0%
Axillary LN L #2							0	14	0%	0	47	0%	11	30	37%	0	16	0%
Axillary LN L #3							0	43	0%	0	5	0%	7	22	32%	0	34	0%
Axillary LN L #4																0	25	0%
Axillary LN L #5																4	40	10%
Axillary LN L #6																0	5	0%
Axillary LN L #7																0	10	0%
Inguinal LN R #1	0	12	0%	9	90	10%	0	11	0%	0	15	0%	6	16	38%	1	27	4%
Inguinal LN R #2	0	15	0%				0	16	0%	0	49	0%	0	10	0%	0	17	0%
Inguinal LN R #3	0	24	0%				0	18	0%	0	23	0%				0	15	0%
Inguinal LN R #4	0	12	0%							0	25	0%				0	16	0%
Inguinal LN R #5	0	19	0%													0	23	0%
Inguinal LN R #6																0	22	0%
Inguinal LN L #1	0	15	0%				0	6	0%				2	12	17%	0	34	0%
Inguinal LN L #2	0	15	0%										1	18	6%	1	14	7%
Inguinal LN L #3	0	17	0%										1	8	13%	0	28	0%
Inguinal LN L #4	0	3	0%													0	38	0%
Inguinal LN L #5	0	24	0%													0	30	0%
Inguinal LN L #6	0	18	0%													0	15	0%
Inguinal LN L #7	0	20	0%													0	36	0%
Inguinal LN L #8																0	14	0%
Inguinal LN L #9																0	13	0%
Inguinal LN L #10																2	25	8%
Inguinal LN L #11																		
Popliteal LN R#1	0	11	0%	2	22	9%	0	8	0%	0	10	0%	0	4	0%	0	5	0%
Popliteal LN R#2																		
Polpiteal LN L #1							0	19	0%	0	5	0%	0	20	0%	0	10	0%
Polpiteal LN L #2							0	1	0%							0	12	0%
Mesenteric LN #1	17	47	36%	15	48	31%				0	16	0%	0	16	0%	6	27	22%
Mesenteric LN #2	0	43	0%	6	30	20%				0	22	0%	0	35	0%	4	14	29%
Mesenteric LN #3	1	55	2%	14	30	47%				0	3	0%	1	10	10%	0	12	0%
Mesenteric LN #4	18	31	58%	9	60	15%				0	49	0%	3	39	8%	2	8	25%
Mesenteric LN #5	0	25	0%							0	12	0%	0	22	0%	5	14	36%
Mesenteric LN #6	0	16	0%							0	16	0%	1	10	10%	0	20	0%
Mesenteric LN #7	8	35	23%							0	19	0%	1	52	2%	5	20	25%
Mesenteric LN #8	1	48	2%										0	17	0%	3	22	14%
Mesenteric LN #9	0	11	0%										1	25	4%	2	17	12%
Mesenteric LN #10	0	22	0%										5	25	20%	1	52	2%
Mesenteric LN #11	0	16	0%										3	9	33%	0	8	0%
Mesenteric LN #12	0	20	0%										2	20	10%	0	47	0%
Mesenteric LN #13	0	10	0%													0	26	0%
Mesenteric LN #14	0	14	0%													0	18	0%
Mesenteric LN #15	3	27	11%													0	24	0%
Mesenteric LN #16	0	16	0%													0	6	0%
Mesenteric LN #17																0	10	0%
Mesenteric LN #18																0	8	0%
Mesenteric LN #19																0	12	0%
Mesenteric LN #20																0	9	0%
Mesenteric LN #21																3	15	20%
Mesenteric LN #22																3	19	16%
Mesenteric LN #23																2	22	9%
Mesenteric LN #24																2	27	7%
Mesenteric LN #25																0	12	0%
Mesenteric LN #26																0	15	0%
Mesenteric LN #27																0	15	0%
Mesenteric LN #28																0	17	0%
Mesenteric LN #29																0	24	0%
Mesenteric LN #30																0	10	0%
Mesenteric LN #31																0	20	0%
Retroperitoneal LN #1	0	27	0%															
Retroperitoneal LN #2	0	30	0%															
Retroperitoneal LN #3	0	12	0%															
Retroperitoneal LN #4																		
Retroperitoneal LN #5																		
Total follicles	68	999	7%	232	1020	23%	0	207	0%	0	455	0%	116	628	18%	70	1713	4%
			0_2-58%			0_6-50%									0_2-78%			0_2-36%
	Positive	Total		Positive	Total		Positive	Total		Positive	Total		Positive	Total		Positive	Total	
Lymph Nodes	9	43	21%	15	18	83%	0	12	0%	0	22	0%	24	31	77%	24	76	32%
Spleen (O.D.) Cut-off = 0.100			0.040			1.790			0.040			0.180			2.720			0.320

Primate Identity	#19			#20			#21			#22			#23			#24		
	Positive follicles	Total follicles	% positive	Positive follicles	Total follicles	% positive	Positive follicles	Total follicles	% positive	Positive follicles	Total follicles	% positive	Positive follicles	Total follicles	% positive	Positive follicles	Total follicles	% positive
**Tonsil #1**	30	33	**91%**	10	12	**83%**	0	22	**0%**							2	10	**20%**
**Tonsil #2**	17	17	**100%**				0	26	**0%**									
**Tonsil #3**																		
**Tonsil #4**																		
**Tonsil #5**																		
**Tonsil #6**																		
**Tonsil #7**																		
**Tonsil #8**																		
**Cervical LN #1**	14	20	**70%**	0	27	**0%**							0	20	**0%**	0	34	**0%**
**Cervical LN #2**				14	35	**40%**										11	45	**24%**
**Cervical LN #3**				25	36	**69%**												
**Cervical LN #4**				9	19	**47%**												
**Cervical LN #5**				13	25	**52%**												
**Cervical LN #6**																		
**Submandibular LN R #1**	17	28	**61%**	17	28	**61%**	0	50	**0%**	0	10	**0%**	0	66	**0%**	5	44	**11%**
**Submandibular LN R #2**	12	25	**48%**	29	49	**59%**							0	54	**0%**	3	12	**25%**
**Submandibular LN R #3**	6	19	**32%**	15	28	**54%**												
**Submandibular LN R #4**																		
**Submandibular LN L #1**	3	11	**27%**	8	23	**35%**	0	11	**0%**	0	25	**0%**	0	35	**0%**	0	9	**0%**
**Submandibular LN L #2**	7	19	**37%**	2	6	**33%**	0	25	**0%**	0	9	**0%**	0	40	**0%**			
**Submandibular LN L #3**				11	30	**37%**							0	25	**0%**			
**Submandibular LN L #4**																		
**Axillary LN R #1**	13	26	**50%**	11	31	**35%**	0	12	**0%**	0	6	**0%**	0	96	**0%**	1	7	**14%**
**Axillary LN R #2**				51	56	**91%**	0	12	**0%**				0	15	**0%**	0	20	**0%**
**Axillary LN R #3**				27	41	**66%**	0	18	**0%**									
**Axillary LN R #4**				1	21	**5%**												
**Axillary LN R #5**																		
**Axillary LN L #1**	1	5	**20%**	28	31	**90%**	0	16	**0%**	0	27	**0%**	0	76	**0%**	0	17	**0%**
**Axillary LN L #2**	4	13	**31%**	11	24	**46%**				0	2	**0%**	0	5	**0%**	5	31	**16%**
**Axillary LN L #3**	1	6	**17%**	3	18	**17%**												
**Axillary LN L #4**																		
**Axillary LN L #5**																		
**Axillary LN L #6**																		
**Axillary LN L #7**																		
**Inguinal LN R #1**	9	11	**82%**	6	40	**15%**	0	4	**0%**	0	37	**0%**	0	20	**0%**	0	29	**0%**
**Inguinal LN R #2**	3	10	**30%**	0	5	**0%**	0	7	**0%**	0	26	**0%**				1	43	**2%**
**Inguinal LN R #3**	20	42	**48%**	5	25	**20%**	0	23	**0%**	0	11	**0%**				1	12	**8%**
**Inguinal LN R #4**	20	58	**34%**	2	25	**8%**												
**Inguinal LN R #5**	2	7	**29%**	0	2	**0%**												
**Inguinal LN R #6**				3	30	**10%**												
**Inguinal LN L #1**	12	15	**80%**	0	20	**0%**	0	18	**0%**	0	6	**0%**	0	29	**0%**	0	11	**0%**
**Inguinal LN L #2**	2	9	**22%**	0	8	**0%**				0	12	**0%**	0	70	**0%**	0	8	**0%**
**Inguinal LN L #3**	13	30	**43%**	0	12	**0%**				0	3	**0%**	0	44	**0%**	0	6	**0%**
**Inguinal LN L #4**	19	50	**38%**	25	48	**52%**										0	6	**0%**
**Inguinal LN L #5**	7	21	**33%**	10	33	**30%**												
**Inguinal LN L #6**	12	32	**38%**	0	37	**0%**												
**Inguinal LN L #7**				0	4	**0%**												
**Inguinal LN L #8**				0	6	**0%**												
**Inguinal LN L #9**				0	13	**0%**												
**Inguinal LN L #10**				0	14	**0%**												
**Inguinal LN L #11**				0	11	**0%**												
**Popliteal LN R#1**				0	11	**0%**												
**Popliteal LN R#2**																		
**Polpiteal LN L #1**				0	11	**0%**	0	6	**0%**									
**Polpiteal LN L #2**																		
**Mesenteric LN #1**	12	27	**44%**	3	30	**10%**	0	14	**0%**	0	8	**0%**	0	13	**0%**	5	54	**9%**
**Mesenteric LN #2**	5	20	**25%**	16	18	**89%**	0	14	**0%**	0	35	**0%**	0	24	**0%**	0	7	**0%**
**Mesenteric LN #3**	3	19	**16%**	18	29	**62%**	0	14	**0%**				0	14	**0%**			
**Mesenteric LN #4**	9	28	**32%**	11	18	**61%**	0	20	**0%**				0	18	**0%**			
**Mesenteric LN #5**	3	30	**10%**	7	9	**78%**	0	5	**0%**				0	35	**0%**			
**Mesenteric LN #6**				3	12	**25%**	0	17	**0%**									
**Mesenteric LN #7**				0	15	**0%**	0	8	**0%**									
**Mesenteric LN #8**				13	13	**100%**												
**Mesenteric LN #9**				0	17	**0%**												
**Mesenteric LN #10**				17	29	**59%**												
**Mesenteric LN #11**				5	25	**20%**												
**Mesenteric LN #12**																		
**Mesenteric LN #13**																		
**Mesenteric LN #14**																		
**Mesenteric LN #15**																		
**Mesenteric LN #16**																		
**Mesenteric LN #17**																		
**Mesenteric LN #18**																		
**Mesenteric LN #19**																		
**Mesenteric LN #20**																		
**Mesenteric LN #21**																		
**Mesenteric LN #22**																		
**Mesenteric LN #23**																		
**Mesenteric LN #24**																		
**Mesenteric LN #25**																		
**Mesenteric LN #26**																		
**Mesenteric LN #27**																		
**Mesenteric LN #28**																		
**Mesenteric LN #29**																		
**Mesenteric LN #30**																		
**Mesenteric LN #31**																		
**Retroperitoneal LN #1**							0	16	**0%**				0	20	**0%**			
**Retroperitoneal LN #2**													0	53	**0%**			
**Retroperitoneal LN #3**													0	20	**0%**			
**Retroperitoneal LN #4**																		
**Retroperitoneal LN #5**																		
**Total follicles**	**276**	**631**	**44%**	**429**	**1110**	**39%**	**0**	**358**	**0%**	**0**	**217**	**0%**	**0**	**792**	**0%**	**34**	**405**	**8%**
			**10-100%**			**0_5-100%**												**0_2-25%**
	**Positive**	**Total**		**Positive**	**Total**		**Positive**	**Total**		**Positive**	**Total**		**Positive**	**Total**		**Positive**	**Total**	
**Lymph Nodes**	**28**	**28**	**100%**	**33**	**49**	**67%**	**0**	**22**	**0%**	**0**	**14**	**0%**	**0**	**22**	**0%**	**9**	**19**	**47%**
**Spleen (O.D.) Cut-off = 0.100**			**1.810**			**1.590**			**0.040**			**0.040**			**0.040**			**2.300**

Primate Identity	#25			#26			#27		
	Positive follicles	Total follicles	% positive	Positive follicles	Total follicles	% positive	Positive follicles	Total follicles	% positive
**Tonsil #1**	0	20	0%	0	28	0%	0	11	0%
**Tonsil #2**	0	14	0%	0	30	0%	0	19	0%
**Tonsil #3**							0	29	0%
**Tonsil #4**							0	26	0%
**Tonsil #5**							0	18	0%
**Tonsil #6**							0	38	0%
**Tonsil #7**							0	20	0%
**Tonsil #8**							0	40	0%
**Cervical LN #1**							0	23	0%
**Cervical LN #2**							0	10	0%
**Cervical LN #3**									
**Cervical LN #4**									
**Cervical LN #5**									
**Cervical LN #6**									
**Submandibular LN R #1**	0	18	0%				0	14	0%
**Submandibular LN R #2**							0	28	0%
**Submandibular LN R #3**							0	28	0%
**Submandibular LN R #4**									
**Submandibular LN L #1**	0	28	0%				0	10	0%
**Submandibular LN L #2**	0	54	0%				0	20	0%
**Submandibular LN L #3**							0	16	0%
**Submandibular LN L #4**									
**Axillary LN R #1**	0	5	0%				0	16	0%
**Axillary LN R #2**	0	7	0%				0	8	0%
**Axillary LN R #3**	0	27	0%						
**Axillary LN R #4**	0	6	0%						
**Axillary LN R #5**									
**Axillary LN L #1**				0	7	0%	0	27	0%
**Axillary LN L #2**				0	7	0%			
**Axillary LN L #3**				0	17	0%			
**Axillary LN L #4**				0	17	0%			
**Axillary LN L #5**				0	10	0%			
**Axillary LN L #6**				0	15	0%			
**Axillary LN L #7**				0	20	0%			
**Inguinal LN R #1**	0	5	0%				0	31	0%
**Inguinal LN R #2**							0	16	0%
**Inguinal LN R #3**							0	21	0%
**Inguinal LN R #4**							0	3	0%
**Inguinal LN R #5**									
**Inguinal LN R #6**									
**Inguinal LN L #1**				0	12	0%			
**Inguinal LN L #2**				0	19	0%			
**Inguinal LN L #3**				0	25	0%			
**Inguinal LN L #4**				0	13	0%			
**Inguinal LN L #5**				0	14	0%			
**Inguinal LN L #6**				0	16	0%			
**Inguinal LN L #7**									
**Inguinal LN L #8**									
**Inguinal LN L #9**									
**Inguinal LN L #10**									
**Inguinal LN L #11**									
**Popliteal LN R#1**							0	12	0%
**Popliteal LN R#2**									
**Polpiteal LN L #1**				0	10	0%			
**Polpiteal LN L #2**									
**Mesenteric LN #1**	0	5	0%				0	5	0%
**Mesenteric LN #2**	0	15	0%				0	16	0%
**Mesenteric LN #3**	0	16	0%				0	7	0%
**Mesenteric LN #4**	0	17	0%				0	42	0%
**Mesenteric LN #5**	0	37	0%				0	8	0%
**Mesenteric LN #6**							0	14	0%
**Mesenteric LN #7**							0	22	0%
**Mesenteric LN #8**							0	4	0%
**Mesenteric LN #9**							0	20	0%
**Mesenteric LN #10**									
**Mesenteric LN #11**									
**Mesenteric LN #12**									
**Mesenteric LN #13**									
**Mesenteric LN #14**									
**Mesenteric LN #15**									
**Mesenteric LN #16**									
**Mesenteric LN #17**									
**Mesenteric LN #18**									
**Mesenteric LN #19**									
**Mesenteric LN #20**									
**Mesenteric LN #21**									
**Mesenteric LN #22**									
**Mesenteric LN #23**									
**Mesenteric LN #24**									
**Mesenteric LN #25**									
**Mesenteric LN #26**									
**Mesenteric LN #27**									
**Mesenteric LN #28**									
**Mesenteric LN #29**									
**Mesenteric LN #30**									
**Mesenteric LN #31**									
**Retroperitoneal LN #1**									
**Retroperitoneal LN #2**									
**Retroperitoneal LN #3**									
**Retroperitoneal LN #4**									
**Retroperitoneal LN #5**									
**Total follicles**	**0**	**274**	**0%**	**0**	**260**	**0%**	**0**	**622**	**0%**
	**Positive**	**Total**		**Positive**	**Total**		**Positive**	**Total**	
**Lymph Nodes**	**0**	**15**	**0%**	**0**	**16**	**0%**	**0**	**33**	**0%**
**Spleen (O.D.) Cut-off = 0.100**			**0.040**			**0.040**			**0.040**
