# Supplementary material for: Heterogeneous transfusion risk linked to variant Creutzfeldt-Jakob disease in a non-human primate model
Source: PLoS Pathog. 2026 Nov 10;22(11):e1014769. doi: 10.1371/journal.ppat.1014769 (PMC13766618; doi:10.1371/journal.ppat.1014769)
Supplement: Supplementary file 3 [file ppat.1014769.s008.docx]

| **Primate donors** | | **Plasma samples** | | | | | | | **Buffy coat samples** | | | | | | |
| --- | --- | --- | --- | --- | --- | --- | --- | --- | --- | --- | --- | --- | --- | --- | --- |
| **Primate** | **Disease phenotype** | PMCA (round +) | **Mice recipients** | | | | | | PMCA (round +) | **Mice recipients** | | | | | |
|  |  |  | Total | Animals with clinical signs | | | | |  | Total | Animals with clinical signs | | | | |
|  |  |  |  | PrP^res^+ vCJD | PrP^res^- incomplete phenotypes | | | |  |  | PrP^res^+ vCJD | PrP^res^- incomplete phenotypes | | | |
|  |  |  |  |  | All | *Brainstem* | *Brain* | *Spinal cord* |  |  |  | All | *Brainstem* | *Brain* | *Spinal cord* |
| #3 | vCJD | +++ | 30 | 0 | 4 | - | 2 | 1 | +++ | 20 | 1 | 1 | 1 | - | - |
| #9 | vCJD | +++ | 20 | 1 | 2 | 1 | - | 1 | +++ | 20 | 0 | 4 | - | 2 | 1 |
| #10 | vCJD | +++ | 20 | 2 | 1 | - | 1 | - | +++ | nd |  |  |  |  |  |
| #11 | vCJD | +++ | 28 | 0 | 3 | 1 | 2 | - | + | 20 | 0 | 0 | - | - | - |
| #12 | vCJD | ++ | 30 | 0 | 2 | - | 2 | - | ++ | nd |  |  |  |  |  |
| #14 | vCJD | +++ | 20 | 0 | 2 | 1 | - | - | +++ | 18 | 0 | 0 | - | - | - |
| #8 | Myelopathy | +++ | 28 | 0 | 3 | 2 | - | 1 | +/- | 20 | 0 | 0 | - | - | - |
| #20 | Myelopathy | +++ | 39 | 0 | 6 | 1 | 3 | 2 | +++ | 20 | 0 | 0 | - | - | - |
| #27 | Myelopathy | - | 24 | 0 | 1 | - | - | 1 | - | nd |  |  |  |  |  |
|  |  |  |  |  |  |  |  |  |  |  |  |  |  |  |  |
| All vCJD primates merged | |  | 148 | 3  (2%) | 14 (9.5%) |  |  |  |  | 78 | 1  (1,3%) | 5 (6,5%) |  |  |  |
| All myelopathic primates merged | |  | 91 | 0 | 10 (11%) |  |  |  |  | 40 | 0 | 0 |  |  |  |
