# Supplementary material for: Heterogeneous transfusion risk linked to variant Creutzfeldt-Jakob disease in a non-human primate model
Source: PLoS Pathog. 2026 Nov 10;22(11):e1014769. doi: 10.1371/journal.ppat.1014769 (PMC13766618; doi:10.1371/journal.ppat.1014769)
Supplement: Supplementary file 4 [file ppat.1014769.s009.docx]

|  | **Duration** | | **Time of sampling** | | | **Plasma PMCA** | | |  | **Buffy coat PMCA** | | |
| --- | --- | --- | --- | --- | --- | --- | --- | --- | --- | --- | --- | --- |
| **Primate #** | **Incubation** | **Clinical period** | **versus injection** | **versus clinical onset** | **% survival** | **Round 2** | **Round 3** | **Round 4** |  | **Round 3** | **Round 4** | **Round 5** |
| 1 | 30 | 3 | 33 | 2 | 100% | 2/2 | nd | nd |  | 0/4 | 4/4 | nd |
| 2 | 47 | 4 | 49 | 3 | 96% | 2/2 | nd | nd |  | 4/4 | 4/4 | nd |
| 3 | 42,5 | 9 | 6 | -37 | 12% |  |  |  |  | 0/4 | 0/4 | 0/4 |
|  |  |  | 8 | -35 | 15% | 0/2 | 0/2 | 0/2 |  | 0/4 | 0/4 | 0/4 |
|  |  |  | 11 | -31 | 22% | 0/2 | 0/2 | 0/2 |  | 0/4 | 0/4 | 0/4 |
|  |  |  | 13 | -30 | 25% | 0/4 | 0/4 | 0/4 |  |  |  |  |
|  |  |  | 19 | -24 | 37% | 0/2 | 0/2 | 2/2 |  |  |  |  |
|  |  |  | 51 | 9 | 98% | 2/2 | nd | nd |  | 4/4 | 4/4 | 4/4 |
| 4 | 45,5 | 12 | 6 | -40 | 10% | 0/2 | 0/2 | 2/2 |  | 0/4 | 0/4 | 0/4 |
|  |  |  | 10 | -35 | 18% | 0/2 | 0/2 | 0/2 |  |  |  |  |
|  |  |  | 14 | -32 | 24% | 0/2 | 0/2 | 0/2 |  | 0/4 | 4/4 | 4/4 |
|  |  |  | 17 | -28 | 30% | 0/4 | 0/4 | 0/4 |  | 0/4 | 0/4 | 0/4 |
|  |  |  | 19 | -27 | 33% | 0/2 | 0/2 | 0/2 |  |  |  |  |
|  |  |  | 21 | -25 | 36% | 0/4 | 0/4 | 0/4 |  |  |  |  |
|  |  |  | 27 | -19 | 47% |  |  |  |  | 0/4 | 0/4 | 0/4 |
|  |  |  | 33 | -13 | 57% |  |  |  |  | 0/4 | 0/4 | 0/4 |
|  |  |  | 37 | -9 | 64% | 0/2 | 0/2 | 0/2 |  | 0/4 | 0/4 | 4/4 |
|  |  |  | 57 | 12 | 99% | 0/2 | 2/2 | nd |  | 2/4 | 4/4 | 4/4 |
| 5 | 34,5 | 3 | 8 | -26 | 21% | 0/4 | 0/4 | 0/4 |  |  |  |  |
|  |  |  | 19 | -15 | 51% | 0/4 | 0/4 | 0/4 |  | 0/4 | 0/4 | 0/4 |
|  |  |  | 37 | 3 | 99% | 0/4 | 0/4 | 0/4 |  | 0/4 | 0/4 | 0/4 |
| 6 | 33 | 2,5 | 31 | -2 | 87% | 2/2 | nd | nd |  | 0/4 | 0/4 | 2/4 |
| 7 | 35 | 9 | 43 | 9 | 97% | 0/2 | 0/2 | 0/2 |  | 0/4 | 0/4 | 2/4 |
| 8 | 42 | 3 | 0 | -42 | 0% | 0/2 | 0/2 | 0/2 |  |  |  |  |
|  |  |  | 10 | -32 | 22% | 0/2 | 0/2 | 2/2 |  |  |  |  |
|  |  |  | 23 | -19 | 51% |  |  |  |  | 0/4 | 0/4 | 4/4 |
|  |  |  | 24 | -18 | 53% |  |  |  |  | 0/4 | 0/4 | 0/4 |
|  |  |  | 29 | -13 | 64% | 0/4 | 0/4 | 2/4 |  | 0/4 | 0/4 | 0/4 |
|  |  |  | 44 | 2 | 98% | 2/2 | nd | nd |  | 0/4 | 0/4 | 0/4 |
|  |  |  | 45 | 3 | 100% | 2/2 | nd | nd |  | 4/4 | nd | nd |
| 9 | 47 | 3,5 | 23 | -24 | 46% | 0/2 | 2/2 | nd |  | 0/4 | 2/4 | nd |
|  |  |  | 50 | 3 | 99% | 2/2 | nd | nd |  | 4/4 | 4/4 | 4/4 |
|  |  |  | 50,5 | 3,5 | 100% | 2/2 | nd | nd |  | 4/4 | 4/4 | 4/4 |
| 10 | 33 | 7 | -8 | -41 | -21% | 0/4 | 0/4 | 0/4 |  | 0/4 | 0/4 | 0/4 |
|  |  |  | 2 | -31 | 5% | 0/4 | 0/4 | 0/4 |  | 0/4 | 0/4 | 0/4 |
|  |  |  | 12 | -21 | 31% | 2/2 | nd | nd |  | 4/4 | nd | nd |
|  |  |  | 21 | -12 | 53% | 2/2 | nd | nd |  |  |  |  |
|  |  |  | 32 | -1 | 79% |  |  |  |  | 4/4 | 4/4 | 4/4 |
|  |  |  | 38 | 5 | 94% |  |  |  |  | 4/4 | 4/4 | 4/4 |
|  |  |  | 40 | 7 | 100% | 2/2 | nd | nd |  | 4/4 | 4/4 | 4/4 |
| 11 | 33 | 5 | 38 | 5 | 100% | 2/2 | nd | nd |  | 0/4 | 0/4 | 4/4 |
| 12 | 32 | 6 | -8 | -41 | -22% | 0/2 | 0/2 | 0/2 |  | 0/4 | 0/4 | 0/4 |
|  |  |  | 0 | -32 | 0% | 0/4 | 0/4 | 0/4 |  | 0/4 | 0/4 | 0/4 |
|  |  |  | 6 | -26 | 16% | 0/4 | 0/4 | 0/4 |  |  |  |  |
|  |  |  | 12 | -20 | 33% | 0/4 | 0/4 | 2/4 |  |  |  |  |
|  |  |  | 21 | -11 | 56% | 0/2 | 2/2 | nd |  |  |  |  |
|  |  |  | 32 | 0 | 84% | 0/2 | 2/2 | nd |  | 0/4 | 2/4 | 4/4 |
|  |  |  | 37 | 5 | 97% |  |  |  |  | 0/4 | 4/4 | 4/4 |
|  |  |  | 38 | 6 | 100% | 0/2 | 2/2 | nd |  | 2/4 | 4/4 | 4/4 |
| 13 | 29 | 8 | 0 | -37 | 0% | 0/2 | 0/2 | 0/2 |  |  |  |  |
|  |  |  | 37 | 7 | 100% | 0/2 | 0/2 | 0/2 |  | 0/4 | 0/4 | 0/4 |
| 14 | 50 | 2 | 31 | -19 | 60% | 2/2 | nd | nd |  |  |  |  |
|  |  |  | 37 | -13 | 71% | 2/2 | nd | nd |  | 4/4 | 4/4 | 4/4 |
|  |  |  | 42 | -9 | 79% | 2/2 | nd | nd |  | 4/4 | 4/4 | 4/4 |
|  |  |  | 50 | 0 | 95% | 2/2 | nd | nd |  | 4/4 | 4/4 | 4/4 |
|  |  |  | 52 | 2 | 100% | 2/2 | nd | nd |  | 4/4 | 4/4 | 4/4 |
| 15 | 30 | 2,5 | 31 | 1 | 96% | 0/2 | 2/2 | nd |  | 0/4 | 0/4 | 0/4 |
| 16 | 30 | 2 | 32 | 2 | 100% | 2/2 | nd | nd |  | 4/4 | 4/4 | 4/4 |
| 17 | 62 | 7 | 24 | -41 | 35% | 0/2 | 2/2 | nd |  | 4/4 | nd | nd |
|  |  |  | 30 | -35 | 44% | 0/2 | 0/2 | 0/2 |  | 4/4 | nd | nd |
|  |  |  | 38 | -28 | 55% | 0/2 | 2/2 | nd |  | 3/4 | nd | nd |
|  |  |  | 42 | -24 | 60% | 2/2 | nd | nd |  | 4/4 | nd | nd |
|  |  |  | 47 | -18 | 69% | 0/2 | 2/2 | nd |  | 4/4 | nd | nd |
|  |  |  | 51 | -14 | 75% | 0/2 | 2/2 | nd |  | 4/4 | nd | nd |
|  |  |  | 57 | -9 | 82% | 2/2 | nd | nd |  | 4/4 | nd | Nd |
|  |  |  | 69 | 7 | 100% | 2/2 | nd | nd |  | 4/4 | 4/4 | 4/4 |
| 18 | 60 | 1 |  |  | 0% | 0/2 | 0/2 | 0/2 |  |  |  |  |
|  |  |  | 61 | 1 | 100% | 0/4 | 4/4 | nd |  | 4/4 | 4/4 | 4/4 |
| 19 | 43 | 5 | 24 | -19 | 51% | 2/2 | nd | nd |  | 4/4 | 4/4 | 4/4 |
|  |  |  | 38 | -6 | 78% | 2/2 | nd | nd |  | 4/4 | 4/4 | 4/4 |
|  |  |  | 42 | -1 | 87% | 0/2 | 2/2 | nd |  | 4/4 | 4/4 | 4/4 |
|  |  |  | 48 | 5 | 100% | 2/2 | nd | nd |  | 4/4 | 4/4 | 4/4 |
| 20 | 40 | 3,5 | 43 | 3 | 100% | 2/2 | nd | nd |  | 4/4 | 4/4 | 4/4 |
| 21 | 32 | 4,5 | 36 | 4 | 100% | 0/2 | 0/2 | 0/2 |  | 0/4 | 0/4 | 0/4 |
| 22 | 141 | 5 | 146 | 5 | 100% | 0/2 | 0/2 | 0/2 |  | 0/4 | 0/4 | 0/4 |
| 23 | 139 | 30 | 169 | 30 | 100% | 0/2 | 0/2 | 0/2 |  | 0/4 | 0/4 | 0/4 |
| 24 | 62 | 4,5 | 66 | 4 | 99% | 2/2 | nd | nd |  | 4/4 | 4/4 | 4/4 |
| 25 | 65,5 | 6 | 71 | 5 | 99% | 0/2 | 0/2 | 0/2 |  | 0/4 | 0/4 | 0/4 |
| 26 | 29 | 8 | 37 | 8 | 100% | 0/2 | 0/2 | 0/2 |  | 0/4 | 0/4 | 0/4 |
| 27 | 23 | 0,5 | 23 | 0,5 | 99% | 0/2 | 0/2 | 0/2 |  | 0/4 | 0/4 | 0/4 |
| Healthy #A |  |  |  |  |  | 0/2 | 0/2 | 0/2 |  | 0/4 | 0/4 | 0/4 |
| Healthy #B |  |  |  |  |  | 0/2 | 0/2 | 0/2 |  |  |  |  |
| Healthy #C |  |  |  |  |  | 0/2 | 0/2 | 0/2 |  | 0/4 | 0/4 | 0/4 |
| Healthy #D |  |  |  |  |  | 0/2 | 0/2 | 0/2 |  |  |  |  |
| Healthy #E |  |  |  |  |  | 0/2 | 0/2 | 0/2 |  | 0/4 | 0/4 | 0/4 |
| Healthy #F |  |  |  |  |  | 0/2 | 0/2 | 0/2 |  |  |  |  |
| Healthy #G |  |  |  |  |  | 0/2 | 0/2 | 0/2 |  |  |  |  |
| Healthy #H |  |  |  |  |  | 0/4 | 0/4 | 0/4 |  |  |  |  |
| Healthy #I |  |  |  |  |  | 0/2 | 0/2 | 0/2 |  |  |  |  |
| Healthy #J |  |  |  |  |  |  |  |  |  | 0/4 | 0/4 | 0/4 |
| Healthy #K |  |  |  |  |  |  |  |  |  | 0/4 | 0/4 | 0/4 |
| Healthy #L |  |  |  |  |  | 0/2 | 0/2 | 0/2 |  | 0/4 | 0/4 | 0/4 |
