# Supplementary material for: Heterogeneous transfusion risk linked to variant Creutzfeldt-Jakob disease in a non-human primate model
Source: PLoS Pathog. 2026 Nov 10;22(11):e1014769. doi: 10.1371/journal.ppat.1014769 (PMC13766618; doi:10.1371/journal.ppat.1014769)
Supplement: Supplementary file 5 [file ppat.1014769.s010.docx]

|  | Plasma (1) | Buffy coat (2) |
| --- | --- | --- |
| Volume of sample | 450µL concentrated on plasminogen-coated beads | 5µL direct |
|  |  |  |
| Substrate | Brain of transgenic mice overexpressing the human Met129 PrP (tg650) (3) | Brain of transgenic mice overexpressing ovine A_136_R_154_Q_171_ PrP (tgShXI) (4,5) |
| Number of rounds | 4 | 5 |
| Number of cycles/round | 80 | 96 |
| Nature of cycles | 20 sec. of sonication  29 min. 40 sec. of incubation | 10 sec. of sonication  14min 50 sec. of incubation |
| Temperature of cycles | 37°C | 39.5°C |
| Number of replicates | 2 | 4 |
| Detection | Western blotting (6) with 3F4 (1 µg/ml) monoclonal anti-PrP antibody after PK digestion | Western blotting (2) with Sha-31 (1 µg/ml) (7) and 12B2 (4 µg/ml) (8) monoclonal anti-PrP antibodies |

1.	Bougard D, Brandel JP, Bélondrade M, Béringue V, Segarra C, Fleury H, et al. Detection of prions in the plasma of presymptomatic and symptomatic patients with variant Creutzfeldt-Jakob disease. Sci Transl Med. 2016 Dec 21;8(370):370ra182.

2.	Lacroux C, Comoy E, Moudjou M, Perret-Liaudet A, Lugan S, Litaise C, et al. Preclinical detection of variant CJD and BSE prions in blood. PLoS Pathog. 2014 Jun;10:e1004202.

3.	Beringue V, Herzog L, Reine F, Le Dur A, Casalone C, Vilotte JL, et al. Transmission of atypical bovine prions to mice transgenic for human prion protein. Emerg Infect Dis. 2008 Dec;14:1898–901.

4.	Groschup MH, Buschmann A. Rodent models for prion diseases. Vet Res. 2008 Aug;39(4):32.

5.	Douet JY, Zafar S, Perret-Liaudet A, Lacroux C, Lugan S, Aron N, et al. Detection of infectivity in blood of persons with variant and sporadic Creutzfeldt-Jakob disease. Emerg Infect Dis. 2014 Jan;20:114–7.

6.	Belondrade M, Nicot S, Béringue V, Coste J, Lehmann S, Bougard D. Rapid and Highly Sensitive Detection of Variant Creutzfeldt - Jakob Disease Abnormal Prion Protein on Steel Surfaces by Protein Misfolding Cyclic Amplification: Application to Prion Decontamination Studies. Baskakov IV, editor. PLOS ONE. 2016 Jan 22;11(1):e0146833.

7.	Feraudet C, Morel N, Simon S, Volland H, Frobert Y, Creminon C, et al. Screening of 145 anti-PrP monoclonal antibodies for their capacity to inhibit PrPSc replication in infected cells. J Biol Chem. 2005 Mar 25;280:11247–58.

8.	Langeveld JP, Jacobs JG, Erkens JH, Bossers A, van Zijderveld FG, van Keulen LJ. Rapid and discriminatory diagnosis of scrapie and BSE in retro-pharyngeal lymph nodes of sheep. BMC Vet Res. 2006 Jun 9;2:19.
